# Experimental evolution in biofilm populations

**DOI:** 10.1093/femsre/fuw002

**Published:** 2016-02-18

**Authors:** Hans P. Steenackers, Ilse Parijs, Kevin R. Foster, Jozef Vanderleyden

**Affiliations:** 1 Department of Microbial and Molecular Systems, Centre of Microbial and Plant Genetics, KU Leuven, Leuven 3001, Belgium; 2 Department of Zoology, University of Oxford, Oxford OX1 3PS, UK; 3 Oxford Centre for Integrative Systems Biology, University of Oxford, Oxford OX1 3QU, UK

**Keywords:** biofilm, adaptive diversification, cooperation, experimental evoultion

## Abstract

Biofilms are a major form of microbial life in which cells form dense surface associated communities that can persist for many generations. The long-life of biofilm communities means that they can be strongly shaped by evolutionary processes. Here, we review the experimental study of evolution in biofilm communities. We first provide an overview of the different experimental models used to study biofilm evolution and their associated advantages and disadvantages. We then illustrate the vast amount of diversification observed during biofilm evolution, and we discuss (i) potential ecological and evolutionary processes behind the observed diversification, (ii) recent insights into the genetics of adaptive diversification, (iii) the striking degree of parallelism between evolution experiments and real-life biofilms and (iv) potential consequences of diversification. In the second part, we discuss the insights provided by evolution experiments in how biofilm growth and structure can promote cooperative phenotypes. Overall, our analysis points to an important role of biofilm diversification and cooperation in bacterial survival and productivity. Deeper understanding of both processes is of key importance to design improved antimicrobial strategies and diagnostic techniques.

## Abstract (precis)

This review paper provides an overview of (i) the different experimental models used to study biofilm evolution, (ii) the vast amount of diversification observed during biofilm evolution (including potential causes and consequences) and (iii) recent insights in how growth in biofilms can lead to the evolution of cooperative phenotypes.

## INTRODUCTION

Biofilms are a major form of microbial life in which single or multiple species of bacteria form densely populated communities, typically enclosed in a matrix of secreted polymers (Costerton *et al*. 1995; Hall-Stoodley and Stoodley 2009; Steenackers *et al*. 2012; Hobley *et al*. 2015). Diffusion in the biofilm is limited, which allows gradients to arise and ultimately results in the formation of a spatially structured, heterogeneous environment (Stewart and Franklin 2008; Nadell, Xavier and Foster 2009). The ubiquity of biofilms is attributable to their ability to colonize many biotic and abiotic surfaces and suggests that living a collective life is critical for bacterial existence and evolution (Hall-Stoodley, Costerton and Stoodley 2004). Biofilm-dwelling bacteria are more tolerant to physical and chemical disruptions than planktonic cells and are a cause of major economic loss within industrial and medical sectors (Davies 2003; Ciofu *et al*. 2015).

Experimental evolution is the study of evolutionary changes that occur in an experimental population as a consequence of conditions imposed by the experimenter (Kawecki *et al*. 2012; Barrick and Lenski 2013). It offers the opportunity to study evolutionary processes experimentally in real time as they happen. Bacteria are very well suited to experimental evolution because of their short generation times and the viability of frozen organisms, which allow an experimenter to create a laboratory ‘fossil record’ for later study (Barrick and Lenski 2013). Numerous evolutionary studies have been performed on the planktonic form of bacteria and yeasts, under diverse environmental conditions (Lenski *et al*. 1991; Dunham *et al*. 2002; Nilsson *et al*. 2005; Hegreness *et al*. 2006; Ackermann *et al*. 2007; Lind and Andersson 2008; Cooper and Lenski 2010; Lang *et al*. 2013; Maddamsetti, Lenski and Barrick 2015) and several reviews discussing experimental evolution of planktonic bacteria (Elena and Lenski 2003; Behe 2010; Kawecki *et al*. 2012; Kassen 2014) are available. In contrast, and despite the predominance of biofilm growth in nature, surprisingly few evolution experiments have been performed with biofilm populations (for an overview; see Table 1).

**Table 1.** Overview of experimental models used to study evolution in biofilms.

Model	Description of model	Focus of studies using the model
Static microcosm of *P. fluorescens*	Non-shaking test tube, in which a biofilm mat forms at the broth–air interface	Effect of ecological opportunity on diversification (Rainey and Travisano 1998) and further evolutionary, ecological and genetic studies on this topic (Kassen and Rainey 2004; Fukami *et al*. 2007; Kassen 2009; Spiers 2014 and more*)
Bead transfer model of *B. cenocepacia*	In slowly rotating test tubes, biofilms are formed on plastic beads, which are regularly transferred to new test tubes. Cells must disperse and colonize a new bead in order to be transferred	Long-term evolution and diversification of *B. cenocepacia* and quantification of fitness of evolved variants (Poltak and Cooper 2011) with follow-up studies that unravel the underlying ecological and genetic mechanisms (Traverse *et al*. 2012; Cooper *et al*. 2014; Ellis *et al*. 2015)*
Spotting on solid agar plates	Agar plates with different compositions, in some cases complemented with disks*	Separate studies have used different agar compositions to investigate: diversification of *Comamonas* sp. (Korona *et al*. 1994) and *E. coli* (Perfeito *et al*. 2008; Saint-Ruf *et al*. 2014) the effect of population structure on diversification (Habets *et al*. 2006) genotypic segregation and drift (Hallatschek *et al*. 2007) the effect of founder cell density on cooperation (van Gestel *et al*. 2014) competition between evolved variants (Kim *et al*. 2014; Koch *et al*. 2014) Additionally, growth media that mimic *in vi*vo conditions have been used (Wong, Rodrigue and Kassen 2012; Gomez and Buckling 2013)*
Non-shaking microtiter plate	Biofilms are grown on the bottom or on discs on the bottom of the wells	Analysis of the evolved variants in *E. coli* (Kraigsley and Finkel 2009), *S. aureus* (Savage, Chopra and O'Neill 2013) and *S. pneumonia* (Allegrucci and Sauer 2007)*
Flow models	Biofilms experience flow conditions. Nutrients are continuously provided and dispersed, allowing unlimited growth	Various reactor devices* have been used in separate studies focusing on: evolved variants of *P. aeruginosa* (Boles, Thoendel and Singh 2004; Kirisits *et al*. 2005; McElroy *et al*. 2014; Penterman *et al*. 2014), *S. marcescens* (Koh *et al*. 2007), *S. aureus* (Yarwood *et al*. 2007; Savage, Chopra and O'Neill 2013), *E. coli* (Ponciano *et al*. 2009) and *S. pneumoniae* (Allegrucci and Sauer 2007) evolved interactions between *P. putida* and *Acetobacter* sp. (Hansen *et al*. 2007) the effect of mutator phenotypes (Lujan *et al*. 2011) public goods and cooperation (Drescher *et al*. 2014) antibiotic resistance (Zhang *et al*. 2011)*
*In silico* models	Mathematical modeling of biofilms	Evolution and stabilization of cooperation (Xavier and Foster 2007; Xavier, Martinez-Garcia and Foster 2009; Nadell, Foster and Xavier 2010; Mitri, Xavier and Foster 2011), polymer secretion by the QS system (Nadell *et al*. 2008) and the role of adhesion in biofilm evolution (Schluter *et al*. 2015)*
*In vivo* models	Biofilm isolates from CF patients	Evolution in patient isolates of *P. aeruginosa* (Smith *et al*. 2006; Kohler, Buckling and van Delden 2009; Hoboth *et al*. 2009 Huse *et al*. 2010; Cramer *et al*. 2011; Warren *et al*. 2011; Yang *et al*. 2011) and *B. dolosa* (Lieberman *et al*. 2011)*

* See Table S1(Supporting Information) for more detailed information about the referred studies.

Biofilm evolutionary studies provide important insights into biofilms, such as informing on the course of chronic infections and persistent contaminations, and aiding in the design of therapeutics and disinfectants (Traverse *et al*. 2012; McElroy *et al*. 2014). In addition, biofilm evolution has been used as a tool to study general evolutionary principles (Rainey and Travisano 1998; Kassen 2009; Spiers 2014). The study of biofilms has revealed striking analogies between life within biofilms and other biological systems. Gradients of oxygen in biofilms for example are analogous to light gradients in forests (Kim *et al*. 2014), while spatial heterogeneity within biofilms in general can be considered as the microscopic counterpart of landscape heterogeneity (Hallatschek *et al*. 2007). Moreover, biofilm evolution carries similarities to the development of malignant cancer tissue, where both are evolutionary and ecological systems where asexual cells divide in a structured environment (Martens *et al*. 2011).

In this review, we first discuss the advantages and disadvantages of a number of biofilm models which have been used for experimental evolution. We then organize our discussion according to two major themes that have emerged in the study of biofilm evolution. The first is the study of the rapid diversification often seen during biofilm evolution experiments and how this shapes both individual genotypes and phenotypes, and the biofilm as a whole. The second theme is how growth in biofilms can lead to the evolution of ‘cooperative’ phenotypes where cells, typically of the same genotype, work together as a collective in a manner that can promote both the growth and resilience of biofilms (Xavier and Foster 2007; Nadell, Xavier and Foster 2009; Drescher *et al*. 2014). We do not restrict ourselves to evolution experiments in the narrow sense of the word, but also discuss findings provided by fitness assays, competition experiments and mutational analyses.

## MODELS TO STUDY EVOLUTION IN BIOFILMS

Biofilm formation is a particularly complex process that depends upon the many environmental factors that can influence the matrix composition and general structure of the biofilm (Goller and Romeo 2008). Importantly, as we will discuss, the evolutionary aspects of biofilms also strongly depend on environmental and experimental factors, such as nutrient levels, bacterial strains under study and their evolutionary history, inoculation sizes, flow rate, substrate area, physical disturbance, experiment duration, etc. For example, strains that have a higher level of preadaptation to the laboratory environment were found to undergo less phenotypic diversification in *in vitro* biofilm evolution experiments than clinical isolates without preadaptation (McElroy *et al*. 2014). Inoculation sizes and flow rates have been shown to influence the distribution of public goods within biofilms and as a consequence the evolution of cooperative traits (Drescher *et al*. 2014; van Gestel *et al*. 2014). And nutrient levels have been shown to affect the strength of diversifying selection and as such phenotypic diversification (Penterman *et al*. 2014).

**Figure 1.**
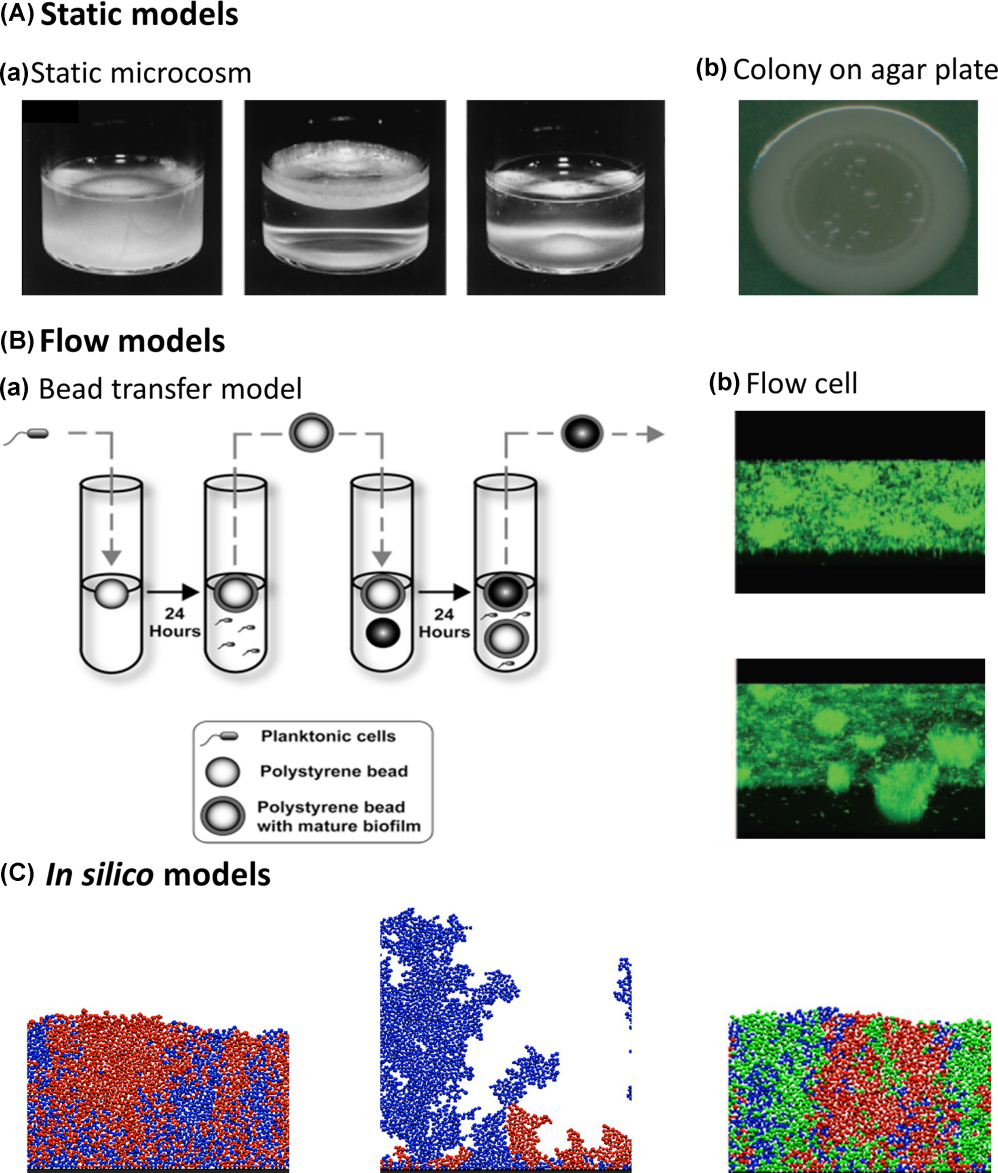
Illustration of biofilm models. (**A)** ‘Static models’**: (**a) The static microcosm model consists of a non-shaking test tube in which a biofilm mat like structure forms at the air–liquid interphase (Adapted from Rainey and Travisano 1998, also used by e.g. Kassen and Rainey 2004; Fukami *et al*. 2007; Kassen 2009; Spiers 2014). (b) Colonies on agar plates are considered to be suitable biofilm models due to the presence of gradients, an increased mutation rate and a structured environment (Adapted from Kim *et al*. 2014, also used by e.g. Korona *et al*. 1994; Perfeito *et al*. 2008; Koch *et al*. 2014; Saint-Ruf *et al*. 2014; van Gestel *et al*. 2014). (B) ‘Flow models’**: (**a) In the bead transfer model, plastic beads are put into slowly rotating test tubes. Biofilms grow on the beads and experience flow conditions due to the rotation of the test tubes. In every transfer cycle, the colonized beads are put in a new tube with new beads, without adding new bacteria (Adapted from Poltak and Cooper 2011, also used by e.g. Poltak and Cooper 2011; Traverse *et al*. 2012; Ellis *et al*. 2015; O'Rourke *et al*. 2015). (b) In flow cells, biofilms can grow in the presence of unlimited nutrients and dispersion (Adapted from Kirisits *et al*. 2005, also used by e.g. Boles, Thoendel and Singh 2004; Hansen *et al*. 2007; Koh *et al*. 2007; Yarwood *et al*. 2007; Lujan *et al*. 2011; Tyerman *et al*. 2013; McElroy *et al*. 2014; Penterman *et al*. 2014; Udall *et al*. 2015). (**C)** ‘*In silico* models’: agent-based *in silico* models consist of single dividing cells (the agents) that are programed to grow until a certain radius and then divide. Different parameters can be easily included and adapted (Adapted from Mitri, Xavier and Foster 2011, also used by e.g. Xavier and Foster 2007; Nadell *et al*. 2008; Nadell, Foster and Xavier 2010; Kim *et al*. 2014; van Gestel *et al*. 2014; Schluter *et al*. 2015).

The experimental model used to study biofilms then can strongly influence what one finds. Moreover, as for much of biology, the study of biofilms faces a strong tension between realism and complexity. While simplicity can be required to identify general evolutionary processes, resemblance to real-life situations is needed to mimic the course of chronic biofilm infections or persistent biofilm contaminations. This tension has led to a wide diversity of biofilm models used in evolutionary studies, and in this section we provide an overview of these models (Fig. 1 and Table 1), together with the associated advantages and disadvantages. A distinction is made between simple static *in vitro* models, *in vitro* flow models, *in silico* models and *in vivo* models. These are only a subset of all existing biofilm models, which have been extensively reviewed before (McBain 2009; Coenye and Nelis 2010; Rumbaugh and Carty 2011).

### Static *in vitro* biofilm models

The simplest *in vitro* model, used in one of the first evolution experiments in biofilms, consists of a glass test tube in which *Pseudomonas fluorescens* is grown under non-shaking conditions (Rainey and Travisano 1998). Due to the static conditions, spatial heterogeneity is created, and the bacteria are able to diversify and form a biofilm-like mat on the surface of the liquid culture. This model thus allows the study of factors driving the formation of biofilms as well as further diversification within biofilms. Other advantages of this model are its simplicity and ease of use, even though a non-shaking test tube is only a valid model for situations where no surface is present, which is for example the case for biofilm-like structures formed in granular sludge in wastewater treatments (Weber *et al*. 2007). Over the years, this *P. fluorescens* radiation in static broth-containing microcosms has become a paradigmatic experimental model for adaptive radiation, and has been applied for numerous evolutionary, ecological and genetic studies on this topic (Kassen and Rainey 2004; Fukami *et al*. 2007; Kassen 2009; Spiers 2014).

In other simple static models, a solid growth medium is used on which evolution in colonies is studied. The conditions in these colonies are considered similar to biofilm conditions. For example when gradients are present, the mutation rate is increased in older colonies, and a spatially structured environment is formed (Bjedov *et al*. 2003). Different growth media have been used including LB agar plates, (Saint-Ruf *et al*. 2014), minimal salt agar (Korona *et al*. 1994), minimal medium (Perfeito *et al*. 2008), modified Schaeffer's medium plates (van Gestel *et al*. 2014), *Pseudomonas* agar F-plates (Kim *et al*. 2014), TSB agar (Koch *et al*. 2014) and the cellulose static disk model (cellulose disks placed on Brain Heart Infusion agar) (Savage, Chopra and O'Neill 2013). In these models, the bacteria are fed from the surface they are growing on and this setup can be a model for biofilms associated with soft tissue infections, cystic fibrosis (CF) or food spoilage (McBain 2009).

Other models have incorporated a hard surface, and the bacteria obtain their nutrients from the liquid culture above the surface. For example, Kraigsley and Finkel (2009) and Boles, Thoendel and Singh (2004) studied evolution in a high-throughput biofilm model consisting of non-shaking polystyrene microtiter plates, in which biofilms grow on the bottom of the wells. This setup can be used for quick screenings, testing numerous different conditions or obtaining a high degree of replication. This model is representative of biofilms on plastic surfaces, such as catheters or food packaging (Hall-Stoodley, Costerton and Stoodley 2004). To study biofilms on additional types of surfaces, small disks composed of different materials may be incorporated into the wells of the microtiter plates, as done with hydroxyapatite plates for the study of oral biofilms (Guggenheim *et al*. 2001).

### *In vitro* flow biofilm models

Many real-life biofilms are exposed to flow conditions and have constant supply of fresh nutrients. To mimic these conditions, flow reactor models can be used. Biofilms that grow under flow conditions are unlimited in their growth, in contrast to static models where nutrients are depleted in time. Also, depending on the flow, varying levels of nutrient dispersion can be achieved (Drescher *et al*. 2014). Different reactor devices have been used in evolution experiments, including drip flow reactors (Boles, Thoendel and Singh 2004; Yarwood *et al*. 2007; Penterman *et al*. 2014), drip-fed columns (Udall *et al*. 2015), tube reactors (Kirisits *et al*. 2005), rotating disk reactors (Boles and Singh 2008), flow cells (Hansen *et al*. 2007; Koh *et al*. 2007; Lujan *et al*. 2011; Tyerman *et al*. 2013; McElroy *et al*. 2014) and the Sorbarod biofilm model (Waite, Struthers and Dowson 2001; Savage, Chopra and O'Neill 2013). A remaining shortcoming is that many models use surfaces and growth media that do not reflect the *in situ* situation. However, flow models can be adapted to specific situations, such as dental plaque biofilms (Spencer *et al*. 2007) or chronic wound biofilms, by introducing other substrates like sliced pork, human tissue or portions of wound dressing (McBain 2009). On the other side, the use of simple surfaces and growth media might also be an advantage, as it allows the study of the effect of different parameters in a simple, defined and homogeneous environment.

Poltak and Cooper (2011) developed an elegant *in vitro* flow model, especially designed for biofilm evolution studies, that introduces the factor of biofilm dispersion. In this model, plastic beads are put into slowly rotating test tubes. Biofilms of *Burkholderia cenocepacia* grow on the beads and experience flow conditions due to the rotation of the test tubes. In every transfer cycle, the colonized beads are put in a new tube with new beads, without adding new bacteria. Thus, only bacteria that are able to attach to the beads in the first tube and disperse from the beads in the second tube are able to colonize the new beads and evolve further. As such, this setup takes into account dispersion in biofilms, colonization of new surfaces and biofilm maturation. Several parameters can be adjusted according to the needs of the experiment. For example, the ratio of colonized beads versus new beads, the time frame and the number of cycles can be changed. As described below, this *B. cenocepacia* bead transfer model has been used in a long-term (∼1500 generations) evolution experiment to unravel the underlying ecological and genetic mechanisms of phenotypic diversification (Poltak and Cooper 2011; Traverse *et al*. 2012; Ellis *et al*. 2015; O'Rourke *et al*. 2015).

### *In vitro* models using growth media that resemble natural conditions

A further development in biofilm models is to use specific growth media that attempt to mimic natural conditions. A first evolution study with *P. aeruginosa* for example simulated the nutritional conditions in a CF lung, by using synthetic CF sputum (Wong, Rodrigue and Kassen 2012). A second study simulated a soil environment to grow *P. fluorescens* under natural conditions, including the presence of the resident microbial community in the soil (Gomez and Buckling 2013).

### *In silico* models

Several *in silico* models have been used especially to study the evolution of cooperation in biofilms. Mainly agent-based models have been applied, in which a single dividing cell is the agent. These models have been developed over the last decade for applications in the field of biochemical engineering and employ mechanistic descriptions of solute diffusion and cell growth (Kreft *et al*. 2001; Picioreanu, Kreft and Van Loosdrecht 2004; Xavier, Picioreanu and van Loosdrecht 2005). Cells are programed to grow depending on the local substrate concentration, extracellular enzyme availability and the concentration of other extracellular products. When a certain radius is met, the cells divide and move until there is no overlap with other cells anymore. Several geometries, including growth on a 2D surface, radial expansions and 3D simulations are possible. The nutrient concentration in the local environment of dividing cells is a function of the nutrient concentration outside the biofilm, the diffusion rate and the consumption by growing cells. At a certain cost, cells may also secrete exopolymeric substances (EPS) or other extracellular products (public goods) such as nutrient scavenging enzymes, which become available to neighboring cells through diffusion and might offer them a benefit. These models have, for example, been applied to determine the outcome of competition (i) between an EPS producing and non-producing strain (Xavier and Foster 2007; Kim *et al*. 2014), (ii) between strains that differ in their EPS production and quorum sensing (QS) phenotype (Nadell *et al*. 2008), (iii) between a public good secretor and non-secretor under different levels of genotypic segregation (Nadell, Foster and Xavier 2010; van Gestel *et al*. 2014), between a public good secretor and a non-secretor in a multispecies biofilm (Mitri, Xavier and Foster 2011) and to study the role of adhesion in biofilm evolution (Schluter *et al*. 2015). An obvious advantage of *in silico* models is that all parameters can be easily adapted. The outcome of such experiments indicates that under certain experimental conditions (combination of parameter values) microorganisms have the ability to behave in a certain way. However, it might not be trivial to link these experimental conditions to real-life situations.

### *In vivo* models

*In vivo* evolution models have mainly focused on *P. aeruginosa* biofilm isolates from CF patients. As CF is associated with an infection that can last for decades, samples from patients are easy to obtain and are invaluable to study bacterial evolution in biofilms *in vivo* (Folkesson *et al*. 2012). Many studies have characterized patient isolates, collected at one time point, which can be used to compare results of *in vitro* evolution experiments with isolated strains (Hogardt and Heesemann 2010; Akers *et al*. 2015). These studies however lack the ability to follow evolutionary dynamics. Nevertheless, a few designed evolution studies have been conducted in which patient isolates were collected over time. Examples include a study of *P. aeruginosa* isolates from the same patient after 6 or 96 months (Smith *et al*. 2006), the identification of mutations in *P. aeruginosa* strains that evolved in patients for 39 000 *in vivo* generations (Huse *et al*. 2010), the characterization of the evolutionary dynamics of *P. aeruginosa* in patients over 200 000 generations (Yang *et al*. 2011), a comparison between mutator and non-mutator strains that evolved in *P. aeruginosa* isolates, taken over 3 yr from 16 patients (Warren *et al*. 2011) and the evolution of QS and virulence in *P. aeruginosa* isolates from 31 patients, collected over 20 days (Kohler, Buckling and van Delden 2009). Whereas the *in vivo* approach provides the most realistic results (Bjarnsholt *et al*. 2013), drawbacks are that it might be difficult to repeat the experiment due to lack of sufficient sample material and to differentiate between adaptive mutations and ‘hitchhiking mutations’ (Wong, Rodrigue and Kassen 2012). Furthermore, many parameters in the *in vivo* experiments are difficult to control due to the heterogeneous conditions. As such, complementation with *in vitro* evolution experiments is very useful. Strikingly, as described below, the same mutations occurring in biofilm *in vitro* evolution experiments can often also be found *in vivo*.

### Multispecies models

Only a few multispecies biofilm evolution experiments have been conducted so far. An *in vitro* two-species evolution experiment was performed with a flow cell biofilm model (Hansen *et al*. 2007). Additionally, evolution in two-species biofilms has been investigated by using the above described *in silico* agent-based model (Mitri, Xavier and Foster 2011).

## DIVERSIFICATION DURING BIOFILM EVOLUTION

A key finding in many biofilm evolution experiments is that the initial microorganisms undergo diversification that is often not observed in planktonic experiments. These biofilm-specific variants can rapidly emerge and remain stable for many generations. In this section, we provide an overview of these diversifications, before discussing the potential ecological, evolutionary and genetic processes causing biofilm diversification. A focus on genetics reveals the remarkable degree of parallelism, both within evolution experiments themselves and between evolution experiments and real-life biofilms. Finally, we discuss the possible consequences of diversification, which include insurance effects, altered productivity, stabilization of cooperative traits and trade-offs between biofilm and free-living conditions.

### Diversification in biofilms is widespread

A growing body of evidence supports the idea that even monospecies biofilms comprise a high level of morphotypic, phenotypic and genotypic heterogeneity. Indeed, in most evolution experiments several genetically stable variants are found to emerge within the first few days of biofilm formation, and these often show striking similarity to variants isolated from real-life biofilms (e.g. Kirisits *et al*. 2005; Smith *et al*. 2006; Cramer *et al*. 2011; Lieberman *et al*. 2011; Traverse *et al*. 2012; Savage, Chopra and O'Neill 2013; Penterman *et al*. 2014).

#### Morphotypic diversity

In the first evolution studies with biofilms, variants were classified based only on their morphotypic appearance. The observed colony morphologies include smooth, wrinkly, fuzzy, sticky, large, small, mucoid, colored and haemolytic. Morphotypic diversification in relation to biofilm evolution was first described by Korona *et al*. (1994) in *Comomonas* sp. and by Rainey and Travisano (1998) in the *P. fluorescens* radiation in static microcosms. In the latter study, three dominant morphotypic variants arose over the course of a few days: the ancestral broth-colonizing smooth morph, the wrinkly spreaders (WS) that form a biofilm mat at the broth–air interface and the fuzzy spreaders that seem to occupy the anoxic zone of the tubes. Later on, morphotypic diversity was also studied in other evolution experiments with amongst others *P. fluorescens* (Gomez and Buckling 2013; Kim *et al*. 2014), *P. aeruginosa* (Deziel, Comeau and Villemur 2001; Boles, Thoendel and Singh 2004; Kirisits *et al*. 2005) and *Staphylococcus aureus* (Yarwood *et al*. 2007).

A remarkable finding is that the timing and pattern of variant formation often follows a predictable sequence, where some variants can only be isolated after other variants emerge. The rise of such successive and dependent variants could either be an effect of epistasis, where the fitness effect of mutations in one gene is dependent upon mutations in other genes, or could—as explained below—be a consequence of modifications in the environment made by earlier variants (niche construction). The evolutionary dynamics of morphotypic diversification has first been explored by Rainey and Travisano (1998), who found that the three dominant morphs in the *P. fluorescens* radiation repeatedly occurred in the same order. Subsequently, Koh *et al*. (2007) showed that evolution of different morphotypes in *Serratia marcescens* biofilms occurred in a time-dependent manner. A defined order of morphotype evolution was also found in *Streptococcus pneumoniae* (Allegrucci and Sauer 2007) and *S. aureus* (Savage, Chopra and O'Neill 2013; Koch *et al*. 2014) biofilms, as well as in the *B. cenocepacia* bead transfer model (Poltak and Cooper 2011), where a smooth (S) variant was consistently identified first at ∼150 generations, followed by a ruffled spreader (R) and wrinkly (W) variant after, respectively, 300 and 300–450 generations.

#### Diversity in other phenotypes

Even though biofilm variants are often first classified by colony morphology, other properties of the evolved variants can also differ strongly, including biofilm formation, dispersion, capsule production, adhesion capacity, antibiotic resistance, antibiotic production, culturability, growth speed and swimming or swarming motility (Boles, Thoendel and Singh 2004; Kirisits *et al*. 2005; Allegrucci and Sauer 2007; Ponciano *et al*. 2009; Koch *et al*. 2014; Penterman *et al*. 2014). Moreover, specific colony characteristics can correlate with biofilm-related traits. For example, the different wrinkly variants isolated by Rainey and Travisano (1998); Boles *et al*. (2004); Poltak and Cooper (2011) in evolution experiments with *P. fluorescens* and *B. cenocepacia* all show increased attachment, increased biomass and increased cluster formation. Similarly, the small colony variants (SCV) isolated by Kirisits *et al*. (2005), Allegrucci and Sauer (2007) and McElroy *et al*. (2014) in *P. aeruginosa* and *S. pneumoniae* all show hyperattachment, increased hydrophobicity, increased biomass and more elaborate 3D biofilm structure. In contrast, the large mucoid (capsular) variants isolated by Allegrucci and Sauer (2007) form flat unstructured biofilms, fail to aggregate in liquid culture and adhere poorly to solid surfaces. However, colonies that are indistinguishable from one another on the basis of colony morphology, also often differ in certain phenotypic assays. For example, the SCVs isolated by Boles *et al*. (2004) showed hyperdetachment and a decreased biomass, in contrast to the earlier described phenotypes that were linked to the SCV morphotype, pointing to a higher underlying complexity and the need for genotypic analyses (Kirisits *et al*. 2005).

#### Genotypic diversity

Early studies on diversification occurred before low-cost sequencing and did not include a genomic analysis of the variants. In the first studies that did incorporate sequencing, the initial classification of the variants was still based on morphological differentiation, and the focus was only on one or two loci thought to be a potential cause of the morphotypic differentiation, based on literature data or suppressor analysis. As a first example, the Rainey group used suppressor analysis and sequencing to show that mutations in the *wsp* locus (which regulates the levels of c-di-GMP and as such production of acetylated cellulose polymer) are responsible for the emergence of mat forming wrinkly spreader (WS) variants in the *P. fluorescens* radiation in static microcosms (Spiers *et al*. 2002, 2003; Goymer *et al*. 2006; Bantinaki *et al*. 2007). To identify additional mutational routes to WS, this approach was repeated on WS strains evolved in an evolution experiment initiated by strains lacking the *wsp* operon, and later also by strains lacking both *wsp* and novel identified loci. This approach revealed two additional loci within which mutation generates the WS phenotype (McDonald *et al*. 2009). Other examples of genes identified by directed approaches include the *cps3D* gene which codes for capsulation in *S. pneumoniae* (Waite, Struthers and Dowson 2001; Allegrucci and Sauer 2007), the accessory gene regulator (*agr*) gene of the QS system in *S. aureus* (Yarwood *et al*. 2007; Koch *et al*. 2014) and the *sigB* gene, coding for the alternative sigma factor in *S. aureus* (Savage, Chopra and O'Neill 2013).

In addition to genotypic characterization of morphotypical variants, the changes in gene expression and the molecular mechanisms underlying phenotypic differentiation have also been studied. Transcriptome analysis was performed for example on *P. aeruginosa* variants and a change in expression of the *psl* and *pel* loci, which have been associated with the adhesion capacity to solid surfaces, was shown to be responsible for the observed phenotypes (Kirisits *et al*. 2005). In addition, the molecular mechanism underlying differences in the culturability of specific variants from *P. aeruginosa* biofilms was studied (Penterman *et al*. 2014).

Whole-genome sequencing and even metagenomic sequencing of evolving populations can now be performed at low cost. This not only allows to further investigate phenotypic variants but also to observe which mutations arise and remain stable without bias to visual variations (Brockhurst, Colegrave and Rozen 2011). A number of studies in this direction have been recently reported. As further discussed throughout the text, whole-genome sequencing was performed for example of phenotypic variants evolved in *P. aeruginosa* biofilms (Wong, Rodrigue and Kassen 2012) and *P. fluorescens* (Kim *et al*. 2014) and *S. aureus* (Koch *et al*. 2014) colonies. With the advent of deep-sequencing protocols, it has become possible to obtain a cross-section of within-population genetic diversity (Traverse *et al*. 2012; Pulido-Tamayo *et al*. 2015). Traverse *et al*. (2012) sequenced DNA from mixed communities, the complete genomes of representative clones and specific alleles of representative clones to reconstruct a nearly complete evolutionary history of long-term diversification in the *B. cenocepacia* bead transfer model. As explained below, striking findings were a recurrent evolution of biofilm specialist morphotypes from generalist types, strong interference competition between contending mutants and multiple adaptive alleles at relatively few loci. Finally, McElroy *et al*. (2014) performed deep sequencing of evolving biofilm populations of two strains of *P. aeruginosa*, 18A and PAO1, at two time points during short-term diversification (5–10 generations).

### Causes of diversification

**Figure 2.**
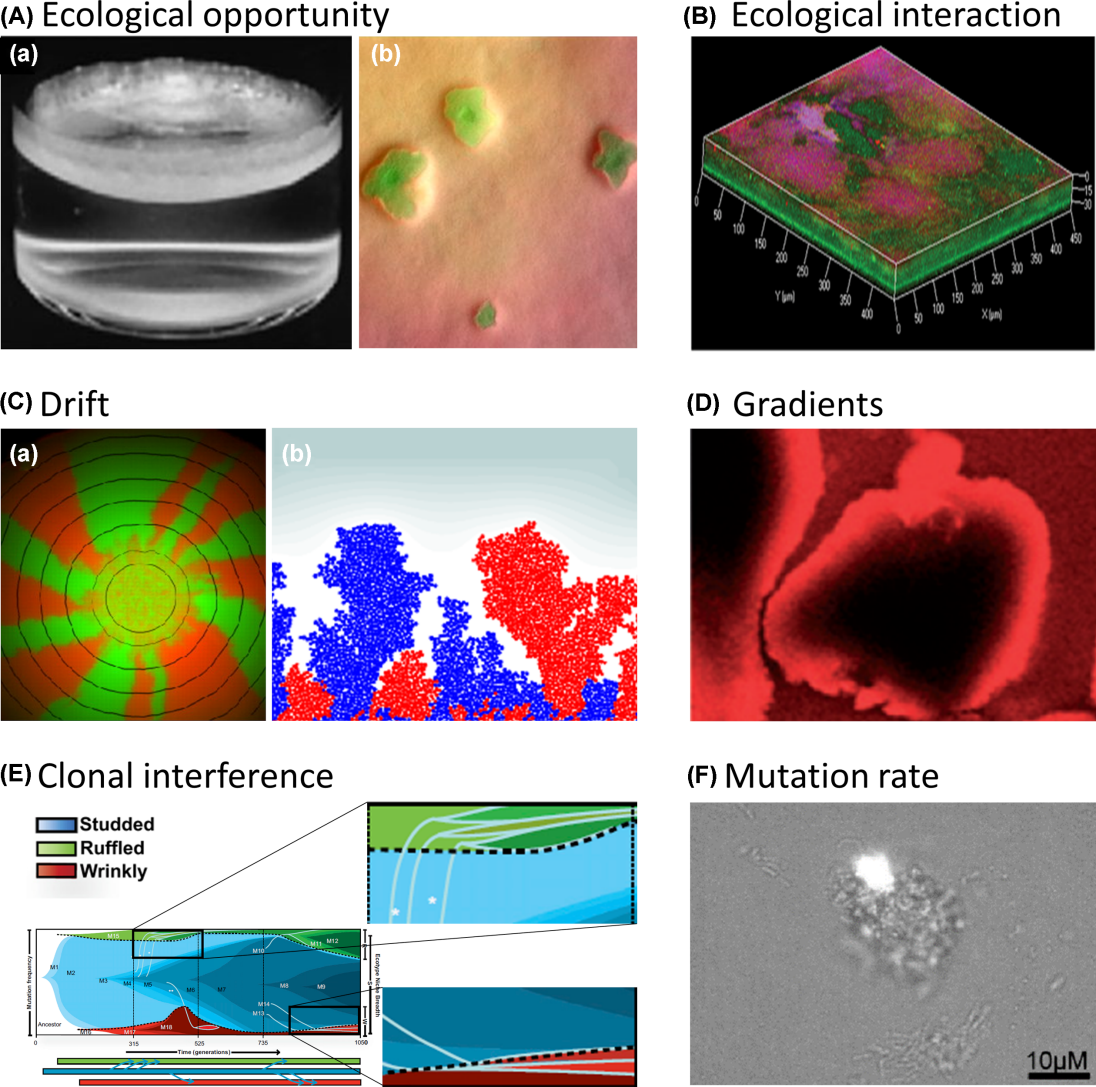
Overview of the causes of diversification. (**A**) ‘Ecological opportunity’: spatial structure and gradients present in a biofilm provide ecological opportunity in the form of vacant niches, which are unused or underutilized by the initially existing genotype(s) and are available to novel genotypes. (a) In a static microcosm, three morphological variants of *P. fluorescens* each occupy a preferred niche: the ancestral SM variant colonizes the broth, the WS variant forms a biofilm mat at the broth–air interphase and the FS seems to occupy the anoxic zone at the bottom of the microcosm (Rainey and Travisano 1998). (b) GFP-tagged MV emerge at the surface when introduced in a wild-type *P. fluorescens* colony. Consistently, MV's that spontaneously arise in *P. fluorescens* colonies push their way to the surface and dominate the colony (Kim *et al*. 2014). (**B)** ‘Ecological interaction’: genotypic variants may alter their environment and consequently create new niches. Ecological succession of the (Studded) S, (Ruffled) R and (Wrinkly) W variants of *B. cenocepacia* is enabled by this process of niche construction. A confocal image of the biofilm structure is shown, in which the entire biofilm is projected in blue, the W morphotype fluoresces red, and the R morphotype fluoresces green. The three endpoint morphotypes were found to partition biofilm space in such a way that the strong biofilm formers R (appearing in yellow) and W (appearing in purple) tightly associate with the beads in heterogeneous clumps and enhance the space available to S, which inhabits a unique layer at the outside of the biofilm on top of R and W (Poltak and Cooper 2011). This spatial segregation was less pronounced at earlier points in evolution (at 350 generations) at which S was found to constitute still a high proportion of the heterogeneous clumps near the bead surface. Thus, the S type appears to have evolved physical displacement from the other types in the biofilms. (**C)** ‘Population structure and drift’: population fragmentation enhances the influence of genetic drift. Drift can be further amplified because often only cells on the expanding edge of the biofilm can grow, which further reduces effective population size. This genetic drift at the expanding frontiers also drives strong population sectoring which can promote the maintenance of diversity. (a) A fluorescent image of a bacterial colony, grown from a mixture of CFP- and YFP-labeled cells reveals spatial segregation of these neutral genetic markers. Only a very thin active layer of growing cells at the boundary of the colony is able to pass on their genes to the next layer of outwardly growing cells, causing a continual bottleneck. The resulting reduction in effective population size promotes a quick segregation of mutants into monoclonal domains (Hallatschek *et al*. 2007). (b) A simulation of surface growth that started with a 1:1 mixture of red and blue cells under low growth substrate conditions. Cell color served as a neutral marker for the lineage segregation in space (Nadell, Foster and Xavier 2010). (**D**) ‘Gradients of stress factors (e.g. antibiotics)’: provide stepping stones that, along with speeding up evolution, also might increase diversification. Indeed, small increases in resistance against the stress factor might be caused by a diversity of mutations of small effect, possibly resulting in a variety of combinations of mutations. Gradients in this biofilm are visualized by imaging the diffusion of the red fluorescent dye rhodamine B into the interior of cell clusters (Rani, Pitts and Stewart 2005; Stewart and Franklin 2008). A movie of the entire sequence can be viewed at https://www.biofilm.montana.edu/resources/movies/2005/2005m01.html. (**E**) ‘Clonal interference’: competition between simultaneous beneficial mutations is expected to lead to longer fixation times and as a consequence to temporally higher diversity. Mutational dynamics within and among niches are shown over time for three evolving morphotypes. Each color transition represents a new haplotype and clonal interference is observed in the case of haplotypes cooccurring within the same niche, for example the haplotypes of the R morphotype (green) (Traverse *et al*. 2012). (**F)** ‘Mutation rate’: mutations are the source of genetic variation for diversifying selection and drift to act on and increased mutation rate enhances the potential for clonal interference, and thus diversity. Mutation frequency in *P. aeruginosa* biofilms is observed using fluorescence (GFP) inducing reversion mutations and clusters of GFP cells within microcolonies are shown (Conibear, Collins and Webb 2009).

Several evolutionary and ecological processes have been proposed to explain diversification in biofilms (Fig. 2), although the relative role of each process remains to be determined.

#### Environmental heterogeneity within biofilms provides ecological opportunity

A biofilm consists of microbial cells that are embedded in a self-produced matrix of extracellular polymeric substances (EPS). The presence of EPS reduces microbial mobility and limits the transfer of chemicals, giving rise to gradients of amongst others nutrients and oxygen (Stewart and Franklin 2008; Nadell, Xavier and Foster 2009). A biofilm is thus a spatially structured and heterogeneous environment, which from an ecological perspective can be seen as a collection of different niches (Chase and Leibold 2003; Kassen and Rainey 2004). A longstanding notion in ecology is the competitive exclusion principle, which states that a single niche can support no more than one genotype (Hardin 1960; Macarthur and Levins 1964). The spatial structure and gradients present in a biofilm provide ecological opportunity in the form of vacant niches, which are unused or underutilized by the the initially existing genotype(s) and are available to novel genotypes (Simpson 1953; Kassen 2009; Yoder *et al*. 2010). Such vacant niches may also exist in free-living populations e.g. when multiple substitutable substrates are present (Barrett, MacLean and Bell 2005), but are expected to be more abundant within biofilms (Habets *et al*. 2006; Kassen 2009).

Theory predicts that diversifying selection, generated by resource competition, favors the emergence of ecological niche specialists, a process called niche partitioning or character displacement (Schluter 2000; Kassen 2009). Ecological specialists trade-off their enhanced competitive advantage in one niche against reduced competitive ability in another. The existence of these trade-offs precludes competitive exclusion by one genotype and makes stable coexistence of genotypes possible (Kassen 2002). One hallmark of this hypothesized mechanism of competitive diversification is negative frequency-dependent selection, which means that the direction of natural selection depends on the frequency of genotypes in such a way that rare types have a fitness advantage. The fitness of the different genotypes changes as a negative function of their frequency, such that variation is maintained (Rainey and Travisano 1998; Brockhurst *et al*. 2006). There is abundant evidence from evolution experiments that the ecological opportunity in biofilms and diversifying selection driven by resource competition play a crucial role in adaptive diversification in biofilms:

- In the *P. fluorescens* radiation in static broth-containing microcosms, a striking relationship was observed between the colony morphotype of the emerging variants and their niche preference: the ancestral smooth morph (SM) variant colonizing the broth, the WS variant, being an EPS hyperproducer and forming a biofilm mat at the broth–air interphase and the fuzzy spreader (FS) seeming to occupy the anoxic zone at the bottom of the microcosm (Rainey and Travisano 1998; Kassen 2009). Later, it was found that the FS in fact forms cellular rafts at the meniscus of the microcosms, which then collapse to the vial bottom and repeatedly reform rafts only to again collapse (Ferguson, Bertels and Rainey 2013). These morphotypes are therefore refered to as ‘ecomorphs’. Moreover, the emergence and maintenance of diversity was shown to require spatially structured (static) microcosms. Indeed, when spatial structure was destroyed by shaking the microcosms, thereby eliminating the multiplicity of niches, diversity was lost from previously diversified populations and did not evolve from genetically uniform ones (Rainey and Travisano 1998; Buckling *et al*. 2000; Kassen, Llewellyn and Rainey 2004; Hall *et al*. 2012). Further, support for the need of ecological opportunity was provided by manipulating the ecological opportunity by inoculating the static microcosms with different combinations of distinct niche specialists prior to introduction of the ancestral smooth morph. The extent of diversification clearly decreased as the ecological opportunity decreased (Brockhurst *et al*. 2007; Gomez and Buckling 2013). An important consequence of this dependency of diversification on prior niche occupation is that immigration history will also influence diversification (Fukami *et al*. 2007). The role of competitive trade-offs was supported by the observation of negative frequency-dependent selection acting in pairwise competitions between the ancestor and derived morphs (Rainey and Travisano 1998). Competition for oxygen was shown to selectively favor the WS variants (Koza *et al*. 2011), which firmly attach to each other and form a self-supporting biofilm mat with superior access to oxygen (Rainey and Travisano 1998; Rainey and Rainey 2003). However, once the mat becomes too heavy it sinks, explaining the frequency-dependent nature of selection (Rainey and Travisano 1998). An important note is that a substantial level of morphological and metabolic variation occurs within each ecomorph (mainly within the WS biofilm mat), which is also associated with a variance in fitness (MacLean, Bell and Rainey 2004; Bantinaki *et al*. 2007; McDonald *et al*. 2009). This intraecomorph diversity rapidly increases over the first few days, after which it is slowly lost (Fukami *et al*. 2007; Meyer *et al*. 2011). This so-called overshooting dynamics was shown to be caused by persistent diversifying selection, which first drives diversification into ecomorphs and intraecomorph variants with distinct resource use, but afterwards leads to loss of ecologically intermediate intraecomorph variants with lower fitness than more extreme types (Meyer *et al*. 2011).
- Where the previous study illustrates the role of spatial heterogeneity, ecological opportunity and diversifying selection in the origin of a biofilm in a static microcosm, similar processes are important as well for evolution inside biofilm communities. Xavier and Foster (2007) used an agent-based model to predict that gradients of oxygen in biofilms can provide a strong selection pressure for copious exopolymer production. This model includes a biochemical description of the carbon fluxes for growth and polymer production, and explicitly calculates diffusion–reaction effects and the resulting solute gradients in the biofilm. It was found that secretion of extracellular polymers by a cell allows it to push descendants up and out into better oxygen conditions. At the same time polymer production harms neighboring non-producers by suffocating them. This effect is analogous to the evolution of woody tissue in plants to grow tall and gain better access to light. Interestingly, under certain conditions, a rare polymer producer can invade a population of non-producers, while the reverse invasion of rare non-producers also happens. This negative frequency-dependent selection predicts a stable coexistence of producers and non-producers and can easily be explained by the suffocation effect, which causes decreasing opportunity for producers to overgrow non-producers when their frequency increases. Although phrased in terms of oxygen gradients, similar results can be expected whenever the biofilm forms a barrier to a limiting resource. Kim *et al*. (2014) provided empirical evidence for this concept, by showing that in colonies of *P. fluorescens* spontaneous mucoid variants (MV) repeatedly arise that push their way to the surface and dominate the colony. The MV do not gain their advantage from a faster intrinsic growth rate, but use secretions to collectively expand and push themselves to the surface of the wild-type colony. Consistently, the MV do not have an advantage in competition with wild-type cells when structure is removed by growing them in liquid cultures or by regularly mixing up the colony.
- Kirisits *et al*. (2005) reported that the isolation of small, wrinkled, sticky colony variants (ST) of *P. aeruginosa* PAO1 from a tube biofilm reactor. Again these variants did not evolve in a shaken liquid culture, where spatial structure was absent. Despite the ST phenotype's increased adherence in short-term adhesion assays, its tendency to stay near the point of initial attachment in a biofilm, and its ability to form large cellular aggregates, it did not predominate the biofilm, but accounted for less than 20% of the population. Even if the biofilm reactor was inoculated with high ST-to-WT ratios, the biofilm always reached a steady-state value around 30%. This observation of negative frequency-dependent selection supports the hypothesis that ST only has a competitive advantage in a particular niche within the biofilm.
- Finally, niche partitioning could also explain the evolution of certain genotypes that at first sight seem to have acquired deleterious mutations. Examples include the stable small non-mucoid variant in a *S. pneumoniae* biofilm, which lacks capsule production, important for the pathogenicity in terms of being able to evade the immune system (Allegrucci and Sauer 2007) and the culture-impaired *P. aeruginosa* variants, which are killed by a self-produced toxin under planktonic conditions (Penterman *et al*. 2014). A possible explanation for why these variants could persist is that they only have an advantage compared to the wild type in a specific niche in the biofilm. Consistent with this idea, these variants did not emerge in shaking liquid cultures.

#### Environmental modification promotes facilitation and ecological succession

Once separated in niche space, the emerging genotypic variants may alter their environment by changing the gradients in existing biotic and abiotic factors or by providing new resources (e.g. metabolic by-products on which new specialists can feed or increased available space), among other factors. This process of environmental modification by organisms has been called ‘niche construction’ (Day, Laland and Odling-Smee 2003) and may increase diversity by providing novel niches available to emerging genotypes (Kassen and Rainey 2004; Kassen 2009; Poltak and Cooper 2011). As such positive, facilitative interactions between genotypes may arise, in which one genotype depends on the presence of the other (Day and Young 2004). Furthermore, changes in the environment can lead to ecological succession (Poltak and Cooper 2011). This phenomenon can be defined using three parameters: (i) the process is orderly, reasonably directional and predictable, (ii) it results from modification of the environment by the community and (iii) it leads to increased productivity for the population (Odum 1975).

- A first piece of data suggesting the importance of facilitation comes from the *P. fluorescens* radiation in static broth-containing microcosms (Rainey and Travisano 1998; Day and Young 2004). As described above, the ability of the three morphotypes to invade a population of a different morphotype from rare was evaluated. All pairwise combinations showed negative frequency-dependent selection, except for the FS type, which cannot invade a population of WS. As such, the ability of FS to emerge after WS in the radiation and their stable coexistence suggest a facilitative interaction of FS with the ancestral smooth type SM.
- A second example indicating the importance of ecological succession has been provided by Poltak and Cooper (2011). In their long-term experiment (1050 generations) of *B. cenocepacia* biofilm propagation using the bead transfer model, three morphotypes evolved in a common pattern of succession: the smooth or studded morphotype (S) at 150 generations, the ruffled spreader (R) at 300 generations and the wrinkly morphotype (W) between 300 and 450 generations. Again the emergence and maintenance of diversity was demonstrated to require spatial structure, since diversity did not evolve in planktonic control cultures and eliminating structure from previously diversified populations by removing the bead significantly reduced diversity. However, positive interactions between the evolving morphotypes were also shown to be important and at the basis of a higher cellular productivity than expected from the productivity of the constituent morphotypes in monoculture:
  - Evaluation of variants isolated from the end of the experiment indicated that cross-feeding plays an important role in this system. Only S is self-sustaining, while neither R nor W can grow in their own supernatants and greatly depend on the metabolic by-products of the other, previously evolved, community members (Poltak and Cooper 2011).
  - The three endpoint morphotypes were found to partition biofilm space in such a way that the strong biofilm formers R and W tightly associate with the beads in heterogeneous clumps and enhance the space available to S, which inhabits a unique layer at the outside of the biofilm on top of R and W (Poltak and Cooper 2011). This spatial segregation was less pronounced at earlier points in evolution (at 350 generation) at which S was found to constitute still a high proportion of the heterogeneous clumps near the bead surface. Thus, the S type appears to have evolved physical displacement from the other types in the biofilms. As expected, this process of niche construction and character displacement was shown to coincide with a reduced competition and enhanced community productivity. Also, trade-offs were associated with niche adaptation since the biofilm output of the late morphotypes inversely correlated with their growth rate (Ellis *et al*. 2015).
    Overall, the observed dynamics may be interpreted as one of ecological succession enabled by niche construction. S likely arose first because it could better exploit the selective environment than its ancestors because of its higher growth rate and biofilm productivity. R and W may have evolved in response by consuming specific metabolites provided by S. Finally, S evolved to occupy a specific biofilm layer, with increased availability of space, provided by R and W.
- Another example is the emergence of six genetically stable morphological variants in a predictable sequence throughout the development of microcolony-type biofilms of *S. marcescens* (Koh *et al*. 2007). The biofilm development progresses through a series of stages during 10 days (formation of microcolonies, emergence of hollow microcolonies, rapid biofilm expansion, cell death and biofilm detachment) and the diversification is strongly correlated with these stages. A sticky-smooth variant (SSV) consistently occurred during hollow microcolony formation, four morphotypes appeared during biofilm expansion, and a smooth-ultramucoid variant (SUMV) (which is derived from SSV) consistently emerged when cell death and biofilm detachment occurred. Moreover, the variants show specialized colonization traits (motility, attachment, biofilm morphology), which are associated with the biofilm stage at which they were isolated. These results again suggest a dynamic of ecological succession in which previous developed morphotypes alter the environment and set the stage for emergence of novel types (Koh *et al*. 2007).

#### Ecological competition causes environmental modification and diversification

Ecological competition between emerging variants can also cause environmental modification. As a response to this competitive environment, strains can further evolve to defend themselves. Koch *et al*. (2014) for instance showed how a single staphylococcal isolate spontaneously diversified by evolving new competitive phenotypes in a coevolutionary arms race. A first evolved strain produced a toxic bacteriocin active against the parent strain. The parent strain counter-adapted to this challenge by generating a second new strain resistant to the bacteriocin.

#### Population structure promotes fixation of diverse mutations of smaller effect

In a spatially structured environment such as a biofilm, the population may be subdivided into a number of more or less independently evolving subpopulations, a phenomenon called population fragmentation (Habets *et al*. 2006). From a theoretical point of view, population fragmentation has two key effects that can lead to enhanced diversity (Habets *et al*. 2006). The first is that small populations experience a higher influence of genetic drift (Wright 1931; Gomez and Buckling 2013; Habets *et al*. 2006). In biofilms, drift can be further amplified because often only cells on the expanding edge of the biofilm can grow, which further reduces effective population size (Golding, Cohen and Ben-Jacob 1999; Hallatschek *et al*. 2007). This genetic drift at the expanding frontiers also drives strong population sectoring which can promote the maintenance of diversity (Klopfstein, Currat and Excoffier 2006; Hallatschek *et al*. 2007; Hallatschek and Nelson 2008). Key to this process is nutrient limitation that ensures that only a subset of the cells in the biofilm can grow at the expanding edge (Nadell, Foster and Xavier 2010; Mitri, Clarke and Foster 2015). A potential second effect of population fragmentation in biofilms is that there can be less access to rare beneficial mutations of large effect. As such, the smaller sub-populations in biofilms may tend to fix beneficial mutations of smaller effect. And because these mutations are more abundant, spatially subdivided populations are likely to follow more diverse adaptive routes (Rozen, de Visser and Gerrish 2002; Rozen *et al*. 2008). Moreover, this fixation of mutations of smaller effect is predicted to slow down the rate of adaptation. Interestingly, the occurrence of slow adaptation and a diversity of adaptive routes may, in the longer term, lead to improved adaptation if it allows the population to discover a rare or complex strategy that would have been lost in a homogeneous fast adapting population (Miralles *et al*. 1999; Nahum *et al*. 2015).

To provide experimental evidence for the role of population fragmentation in biofilm diversification, Habets *et al*. (2006) propagated populations founded by a single genotype of *Escherichia coli* for 900 generations in either a homogeneous environment (shaken liquid culture), a heterogeneous environment with a population structure that was kept intact (colony on agar plate, transferred by a stamp) or a heterogeneous environment in which the population structure was destroyed each day (colony on agar plate, mixed every day before transfer to a new plate). In line with theory, population structure enhanced adaptive radiation, since significant diversity in catabolic activity among evolved clones was only observed in spatially structured environments with intact population structure. Moreover, negative frequency-dependent fitness interactions were found, suggesting that diversity is stably maintained. An interesting note is that the fitness compared to the ancestor was lower for populations evolved in the heterogeneous environment with intact population structure than those with destroyed population structure, as predicted in case of fixation of beneficial mutations of smaller effect. This study did not address whether this heterogeneity can ultimately improve the process of adaptation. However, another recent study using liquid culture did find that population subdivision can improve adaptation in *E. coli* experimental evolution (Nahum *et al*. 2015).

#### Presence of gradients provides stepping stones for diverse mutations of smaller effect

Often several mutations are required for a bacterium to obtain significant resistance to stresses like antibiotics (Lipsitch 2001). In a homogeneous environment, a bacterium has to rapidly acquire all these mutations to be able to survive. The presence of gradients in the stress factors however might provide stepping stones (also called ‘sanctuaries’ or ‘resistance-selective environments’) allowing these mutations to be selected one by one (Baquero *et al*. 1998; Baquero and Coque 2014). Ecologically, these functions by the sanctuary providing a source of immigrants that continually seed the toxic environment giving the population many opportunities to adapt (Perron, Gonzalez and Buckling 2007). Zhang *et al*. (2011) used an ingenious assay to demonstrate that concentration gradients can indeed strongly promote antibiotic resistance development. By means of a complex microfluidic device, a smoothly varying concentration gradient of ciprofloxacin was set up in a two-dimensional landscape of connected hexagonal wells. Remarkably, high resistance, due to four single-nucleotide substitutions in three genes, evolved in only 10 h after inoculation of the *E. coli* bacteria in the center of the device.

Hermsen, Deris and Hwa (2012) developed a mathematical model, called the ‘staircase model’ to explain how antibiotic gradients can promote adaptation. This suggests that the gradients allow resistant mutants to evade competition and circumvent the slow process of fixation by invading compartments with higher drug concentrations, where less resistant strains cannot subsist. Although the role of this mechanism within biofilms remains to be proven, the common presence of gradients within biofilms means it may be relevant in nature (Stewart and Franklin 2008). How could this process, in addition to increasing the rate of adaptation, also increase diversification? First, small increases in resistance might be caused by a diversity of mutations of small effect (Martinez and Baquero 2000), possibly resulting in a variety of combinations of mutations. Second, antibiotic resistance (or stress resistance in general) often requires adaptations that carry a fitness cost in the absence of the antibiotic (Andersson 2006). The staircase model predicts that this fitness trade-off between niches with high and low antibiotic concentration will prevent resistant strains from outcompeting less-resistant strains in regions of lower antibiotic concentration, which may stabilize diversity (Hermsen, Deris and Hwa 2012).

#### Clonal interference increases fixation times and enhances temporal diversity

The traditional view of evolution by natural selection is that adaptation occurs via rare beneficial ‘driver’ mutations, which will sequentially reach fixation via selective sweeps that purge all variation and preserve the clonal genotype (i.e. periodic selection). However, recent evolution experiments have shown that beneficial mutation rates are typically high enough so that multiple driver populations can cooccur. Competition between these alternative beneficial mutations, a phenomenon called clonal interference, is expected to lead to longer fixation times and as a consequence to temporally higher diversity (Greaves and Maley 2012; Barrick and Lenski 2013). Theory suggests that such competition can also produce a simultaneous sweep of multiple linked mutations, because more than one beneficial mutation may be required to prevail in competition (Park and Krug 2007; Sniegowski and Gerrish 2010). *In silico* simulations have predicted that clonal interference is more prevalent with spatial structure than without, due to the slow wave-like spread of beneficial mutations through space in structured populations, compared to the fast Malthusian sweeps in well-mixed populations (Martens and Hallatschek 2011; Martens *et al*. 2011). Consistently, Traverse *et al*. (2012) observed a large prevalence of clonal interference in the *B. cenocepacia* bead transfer model, since new mutants coexisted with each other for hundreds of generations.

#### Increased mutation rate and horizontal gene transfer

An increased mutation rate and horizontal gene transfer (HGT) may also promote diversification in biofilms. Indeed, bacteria in a biofilm can exhibit a 2- to 60-fold increase in mutation rate compared to planktonic cultures (Loewe, Textor and Scherer 2003; Conibear, Collins and Webb 2009; Ryder, Chopra and O'Neill 2012). Mutation rates are historically seen largely invariant and driven by inevitable errors in DNA replication (Drake *et al*. 1998). However, it is now appreciated that bacterial mutation rates and rates of horizontal DNA transfer can increase greatly under certain stresses, with the effect that the rate of adaptation will increase under conditions where a cell is not well adapted to its environment. Additionally, mutational hotspots can occur because stress-induced mutations are linked to local events such as DNA breaks (Galhardo, Hastings and Rosenberg 2007; Rosenberg and Queitsch 2014).

Oxygen stress can be a reason for increased mutation rate in biofilms, caused by deleterious effects on the DNA by reactive oxygen species (ROS) themselves, or by the induction of the ROS stress response. The presence of ROS has been shown to be a source of genotypic, morphotypic and/or phenotypic diversification in *S. pneumoniae* (Allegrucci and Sauer 2007), *P. aeruginosa* (Boles and Singh 2008; Driffield *et al*. 2008; Conibear, Collins and Webb 2009; Starkey *et al*. 2009), *S. aureus* (Ryder, Chopra and O'Neill 2012) and *E. coli* (Saint-Ruf *et al*. 2014) biofilms. Addition of antioxidants or the use of mutants affected in ROS production reduced diversification in these studies; addition of hydrogen peroxide or the use of oxidant hypersensitive mutants increased diversification. A possible explanation for increased oxygen stress in biofilms is the presence of steep gradients, which rapidly generate stress by accumulation of toxic metabolites (Stewart and Franklin 2008; Conibear, Collins and Webb 2009; Saint-Ruf *et al*. 2014). Consistently, genes involved in the oxidative stress response were found to be upregulated in *E. coli* colonies (Saint-Ruf *et al*. 2014) and *S. aureus* biofilms (Ryder, Chopra and O'Neill 2012). On the contrary, in *P. aeruginosa* biofilms it was found that several genes coding for oxidative DNA damage protection enzymes were downregulated (Driffield *et al*. 2008). This imbalance between oxidant burden and antioxidant defense might make the biofilm cells even more susceptible to oxygen stress.

The mechanisms by which ROS induce DNA damage in biofilms are not completely clear. In *E. coli*, ROS can induce the general stress RpoS response and the SOS response, which are required in stress-induced mutagenesis systems. However, Boles and Singh (2008) found none of these responses to be necessary for the generation of variation in their *S. aureus* biofilm model. Instead, it was shown that ROS can cause double-stranded breaks in the DNA, which give rise to genetic variants when they are repaired by a mutagenic mechanism involving combinatorial DNA repair genes (amongst others *recA*) (Rosenberg 2001; Boles, Thoendel and Singh 2004). An interesting study on the relative role of ROS-induced mutation rate and diversifying selection in the generation of morphotypic variation was conducted by Allegrucci and Sauer (2008). Addition of hydrogen peroxide to planktonic *S. pneumomiae* cultures was shown to generate a similar diversity as observed in biofilms: the same morphotypes arose, in the same order and in similar frequencies. These results suggest that at least in this system, morphotypic diversification is not due to selection for variants better equipped for adherence and biofilm growth but instead is likely the result of increased mutation rates induced by oxidative stress conditions. In contrast, Wrande, Roth and Hughes (2008) proved the accumulation of rifampicin-resistant (Rif^R^) mutants, in *Salmonella enterica* and *E. coli* colonies to be caused by selection mechanisms rather than stress-induced mutagenesis, which was previously thought to be the cause of these observed mutations (Taddei, Matic and Radman 1995). In these colonies, the general mutation rate was not enhanced as only an increase in Rif^R^ mutants, which have a growth advantage, was observed (Wrande, Roth and Hughes 2008). Next, to the ROS-mediated increased mutation rate, nutrient limitation can also be a cause of stress-mediated mutations, as observed in *E. coli* biofilms (Ponciano *et al*. 2009). Finally, other stress responses that induce mutations in bacteria have been reviewed by Galhardo, Hastings and Rosenberg (2007).

Mutation rate itself can also evolve independently of stress responses. Indeed, hypermutator phenotypes with an increased mutation rate can emerge. Natural selection can favor these hypermutator strains when adapting to a new environment because the hypermutator genes can hitchhike with beneficial mutations they generate (Metzgar and Wills 2000; Denamur and Matic 2006). In addition, spatial heterogeneity can favour such hypermutators by allowing them to spread to favorable environments should conditions change again (Travis and Travis 2004). Consistent with these ideas, hypermutator phenotypes are found in many biofilms. For example, by studying 90 isolates that were obtained over time from 29 CF patients, it was found that 17% of these strains contained a mutation in the MutS-MutL mismatch repair system, causing a mutator phenotype. Other mutations that had an effect on the virulence and drug efflux pumps were found as well, possibly under influence of the mutator phenotype (Smith *et al*. 2006; Mena *et al*. 2008). In order to better understand the impact of mutator phenotypes, Lujan *et al*. (2011) competed artificially constructed *mutS* mutants of *P. aeruginosa* with the wild type in a flow cell biofilm model and in planktonic phase. The mutators showed an enhanced adaptability over the wild type when grown in biofilms but not as planktonic cells. This advantage was associated with enhanced micro-colony development and biofilm architecture by the mutators, as also seen by Conibear, Collins and Webb (2009), and by a faster and more extensive phenotypic diversification. Similarly, Saint-Ruf *et al*. (2014) observed a faster and more extensive diversification in *E. coli* colonies founded by *mutS* and *mutT* mutator strains compared to those founded by wild-type cells. However, mutator phenotypes are self-limiting due to the high risk of deleterious mutations (Denamur and Matic 2006).

HGT is yet another proposed cause of the increased mutation rate in biofilms, because the HGT rate is often higher in biofilms compared with planktonic cultures, due to a higher conjugation rate, a higher transformation rate, an increased plasmid stability, high cell density, stable environment for physical cell–cell contact and high amount of eDNA present in the matrix (Madsen *et al*. 2012; Burmolle *et al*. 2014). Furthermore, Antonova and Hammer (2011) showed that HGT can be induced by autoinducers in a mixed species biofilm containing *Vibrio cholera*. HGT is thought to be an important process in the acquisition of mutations that induce social phenotypes because genes of social traits can be acquired (Mitri and Foster 2013). Although in most studies the increased HGT is not mentioned as possible explanation, it is highly plausible that this effect is an important cause of diversification in many biofilm evolution experiments. In addition, other possible causes of an increased mutation rate include genomic rearrangements and mobility of insertion sequences (Saint-Ruf *et al*. 2014).

### Genetic mechanisms behind adaptive diversification

The genetic mechanisms underlying adaptive diversification have been studied in most detail in the *P. fluorescence* radiation in static microcosms and the *B. cenocepacia* bead transfer model. These studies also revealed insight into the molecular basis of fitness trade-offs across niches and the competitive dynamics of genotypes within and across niches.

#### Antagonistic pleiotropy in the *P. fluorescens* radiation in static microcoms

The transition from smooth to WS in the *P. fluorescens* radiation in static broth-containing microcosms and the associated invasion of the air–broth interface is caused by the constitutive activation of a pathway for the production of a cellulose-like polymer, encoded by the *wss* operon. The activation of Wss occurs through a range of loss-of-function mutations in the methylesterase WspF (part of the Wsp signal transduction pathway), leading to increased methylation of the Wsp receiver complex and a constitutive activation (through phosphorylation) of the diguanylate cyclase response regulator WspR, resulting in enhanced levels of cyclic diguanosine monophosphate (c-di-GMP) (Spiers *et al*. 2002, 2003; Goymer *et al*. 2006; Bantinaki *et al*. 2007). C-di-GMP is an intracellular signalling molecule that plays a central role in the regulation of motility, virulence and biofilm formation in *P. fluorescens* and many other bacteria (Hengge 2009; Romling, Galperin and Gomelsky 2013). As such enhanced WspR activity results in the constitutive activation of Wss and an increased production of acetylated cellulose needed for forming the biofilm mat (Spiers *et al*. 2003). Interestingly, the genetically distinct mutations leading to the WS morphotype are associated with a diverse array of fitness values, which sets the stage for divergence by selection among independently arisen WS (Bantinaki *et al*. 2007).

The evolution of a diverse community in a heterogeneous environment requires that no single generalist type is able to outcompete all the others in all niches of the environment. The evolution of such generalists is thought to be unlikely, because trade-offs in fitness across environments are expected, either because of antagonistic pleiotropy (alleles beneficial in one environment are deleterious in others) or mutation accumulation (mutations neutral in the environment of selection that disrupt function in novel environments) (Kassen 2009). Antagonistic pleiotropy is clearly implicated in the emergence of the biofilm mat-forming WS in the *P. fluorescens* radiation in static microcosms. Biolog fitness assays and proteome analysis indicated that WS genotypes express catabolic defects, which could be attributed to the same mutations causing the overexpression of acetylated cellulose needed for mat-formation (MacLean, Bell and Rainey 2004; Knight *et al*. 2006). Interestingly, prolonged selection in the spatially structured microcosms caused the cost of adaptation to decline, without loss of the benefits associated with adaptation, an observation which could either be explained by compensatory adaption with specialist lineages or clonal competition among specialist lineages.

#### Competition occurs within and between niches in the *B. cenocepacia* bead transfer model

Metagenome sequencing of the diversifying population in the *B. cenocepacia* bead transfer model revealed that changes in c-di-GMP metabolism are also underlying the emergence of the S, R and W morphotypes in this system and are at the basis of their distinct biofilm attachment patterns (and thus niche preference) (Traverse *et al*. 2012). The initial S and R morphotypes originated from the ancestor by two different SNPs in the *yciR* gene, which encodes both a diguanylate cyclase (synthesizing c-di-GMP) and phosphodiesterase (degrading c-di-GMP) domain. Alternative *yciR* alleles thus define morphological and ecological differences in this system. Finally, the initial W morphotype acquired a mutation in *wspA*, part of the Wsp receiver complex described above. In these, mutations were associated with a strong increase in frequency of each morphotype, and the enhanced access to beneficial mutations resulted in further adaptation of each morphotype. The S lineage for example consequently accumulated (i) a SNP in a TCA cycle enzyme, (ii) a large deletion that removed the previously mutated *yciR* locus, (iv) a single bp deletion in *manC* which promoted biofilm production, (v) a change in the promoter of the iron storage gene bacterioferritin that increased its expression and (vi) a deletion of 49 diverse genes. An interesting finding was that different new mutants coexisted with each other for hundreds of generations (a dynamics called clonal interference), which contrasts with the sequential replacement that often occurs in unstructured environments (periodic selection). As described above this provides an additional source of genetic diversity.

An intriguing observation was that competition between genotypes (clonal interference) did not only occur within niches but also between niches, suggesting a more fluid community structure (Traverse *et al*. 2012). Indeed, the biofilm specialist types R and W did recurrently evolve anew from the single evolving S lineage. Remarkably, a W haplotype that gained a fitness advantage by a mutation in the promoter of bacterioferritin was not able to spread beyond this niche. Instead, new mutants of S with upregulated bacterioferritin evolved as a response and succeeded to invade other niches again. Only the S lineage, which balances fitness between planktonic and biofilm conditions (and can be considered as a generalist) was thus able to spawn new R and W types at least seven times. The S lineage may be more evolvable because of its larger population size, absence of negative epistasis, or larger ecological breadth. As a consequence, the endpoint S, R and W morphotypes share a common adaptive haplotype derived from the early S clone (with mutations in TCA cycle, *yciR, manC* and bacterioferritin) and have only a few mutations distinguishing them from each other. The endpoint R type evolved from S by four mutations, whereas the endpoint W types evolved by single mutations in *wspA* and *wspE*, similar to the original W lineage. Finally, the S lineage experienced a further 49-gene deletion.

### Parallelism in diversification

The increasing amount of reports on parallel evolution in nature and *in vitro* show that evolution can be highly reproducible (Gerstein, Lo and Otto 2012; Meyer *et al*. 2012; Herron and Doebeli 2013; Stern 2013). Also biofilm evolution experiments have indicated a striking amount of parallelism at the morphotypic, phenotypic and genotypic level, both between replicate lineages within the same evolution experiment, between different evolution experiments (performed in different labs) and importantly, between *in vitro* evolution experiments and evolution in chronic infections. The rapidity and repeatability of biofilm evolution are indicative of strong selective pressures within biofilms. The exceptional parallelism found between laboratory-derived variants and *in vivo* isolates indicates that some of the same forces that drive biofilm adaptation *in vitro* also contribute to adaptation during chronic infections.

#### Replicate lineages within the same evolution experiment

In the *P. fluorescens* radiation in static broth-containing microcosms, the three dominant morphological classes (S, WS and FS) showed highly repeatable evolutionary dynamics across replicate populations. However, slight variation was always encountered within the morphological classes across microcosms. The mutational origins of 26 independent WS genotypes were unraveled by suppressor analysis and sequencing (Bantinaki *et al*. 2007; McDonald *et al*. 2009). All mutations reside exclusively in one of three pathways (Wsp, Aws and Mws), each harboring a diguanylate cyclase responsible for the production of c-di-GMP. The majority of mutations were found to cause loss-of-function changes in a few negative regulators of the diguanylate cyclases, as such enhancing the levels of c-di-GMP and acetylated cellulose required to form the biofilm mat. Eliminating the three pathways from the *P. fluorescens* genome and replaying evolution revealed 13 new mutational pathways that allow realization of the WS phenotype. Remarkably, all 13 pathways harbor diguanylate cyclases and overexpression of c-di-GMP and acetylated cellulose is thought to be the cause of the WS phenotype in each case. WS morphotypes with mutations in these new pathways however took longer to arise, which explains why they were not present among the 26 WS genotypes derived from the ancestral strain (Lind, Farr and Rainey 2015). Since these WS types arisen via the new pathways do not differ in fitness relative to the earlier identified WS types, their later appearance cannot be attributed to a selective disadvantage. Genetic constraints, mediated by genetic architecture and the mutational target size, have therefore been proposed to be of crucial importance as well to explain genetic parallel evolution (McDonald *et al*. 2009; Lind, Farr and Rainey 2015). Indeed, certain pathways have a greater capacity than others to translate mutation into phenotypic variation, and a number of principles have been derived from the data: evolution proceeds firstly via pathways subject to negative regulation, then via promoter mutations and gene fusions, and finally via activation by intragenic gain-of-function mutations (Lind, Farr and Rainey 2015).

In the *B. cenocepacia* bead transfer model, each of the six replicate biofilm populations underwent a common pattern of adaptive morphological diversification, in which the three ecologically distinct morphotypes (S, R and W) arose in the same order of succession. Although a detailed evolutionary model was only assembled for one lineage (see above), mutations associated with adaptation were identified by metagenome sequencing in early and late samples of all six lineages. An exceptional parallelism among adaptive targets was observed with at least 26 mutations involved in c-di-GMP metabolism (Seven mutations in *yciR*, 18 mutations in the *wsp* operon), 20 mutations in a gene cluster involved in LPS biosynthesis, and several mutations in the TCA cycle, RNA polymerase subunits *rpoC* and *ropD* and a galactose metabolism operon (Poltak and Cooper 2011; Traverse *et al*. 2012). This parallelism can likely be attributed to strong natural selection. Indeed, metagenome sequencing revealed several features that suggest a strong role for selection in this system as follows. (i) Specific mutations rapidly rose to high frequency (e.g. five mutations became detectable in the S lineage within 315 generations). (ii) Mutations in coding sequences were mostly nonsynonymous, intergenic mutations were associated with likely promoters, and deletions affected genes that were plausible targets of selection. (iii) The rapid rise of the different lineages combined with the low per-genome mutation rate should theoretically hinder hitchhiking of neutral mutations.

McElroy *et al*. (2014) sequenced the metagenome of populations of two *P. aeruginosa* strains (the lab strain PAO1 and the clinical isolate 18A) at two time points during short-term biofilm evolution (∼5.3 and 10.3 generations in total for PAO1 and 18A, respectively). Also, here a strong within-strain parallel evolution was observed, often involving identical nucleotides, which—in agreement with an enhanced mutation rate within biofilms–indicates that the mutation rate was not limiting. In contrast, there was an almost complete lack of non-coding and synonymous mutations, suggesting that the majority of the *P. aeruginosa* genome is constrained by negative selection, with strong positive selection acting on an accessory subset of genes facilitating adaptation to the *in vitro* biofilm lifestyle (McElroy *et al*. 2014). Parallel evolution at the nucleotide level was a striking finding, as this is considered to be a rare event for bacteria (Dettman *et al*. 2012).

Finally, Kim *et al*. (2014) showed that in colonies of *P. fluorescens* spontaneous MV repeatedly arise that produce secretions to push their way to the surface and gain better access to oxygen. Analysis of over 500 independent adaptation events revealed a striking level of parallelism because all of them occurred through mutation of a single repressor of secretions, RsmE. For many positions, the same mutation was even found multiple times, up to a maximum of 69 cases of a particular SNP. Since none of the mutations were synonymous and the mutation rate of *rsmE* does not exceed the genome average, this parallelism was attributed to strong natural selection. Consistently, all mutants were able to outcompete the wild type, although a strong variability in competitive ability among the mutants was observed. Interestingly, generation of a fine-scale map of the mutations provided an explanation for this variability in competitiveness in terms of molecular structure and function.

#### Comparison of different *in vitro* evolution experiments

Colony types with similar characteristics have been isolated in different evolution experiments, even across different bacterial species. SCV were for example isolated by Kirisits *et al*. (2005), Allegrucci and Sauer (2007) and McElroy *et al*. (2014) in *P. aeruginosa* and *S. pneumoniae*. As discussed above, in some cases these SCV also show similarity in other phenotypes, such as hyperattachment, increased biomass and more elaborate 3D biofilm structure. Another example is represented by the wrinkly variants of *P. fluorescens* and *B. cenocepacia* isolated in different evolution experiments by Rainey and Travisano (1998), Boles *et al*. (2004) and Poltak and Cooper (2011), which all show increased attachment, increased biomass and increased cluster formation.

Strong parallelism between experiments was also observed at the genomic and molecular level. Despite the limited number of genomic studies, changes in c-di-GMP metabolism, often mediated by mutations in the *wsp* operon, were found to play a major role in morphotypic and phenotypic differentiation in at least three independent evolution experiments (McDonald *et al*. 2009; Traverse *et al*. 2012; McElroy *et al*. 2014). Additionally, mutations that caused an effect on the LPS due to a mutation in glycosyltransferase genes were found both in *P. fluorescens* (*fuzY*) (Ferguson, Bertels and Rainey 2013), *B. cenocepacia* (*manC*) (Traverse *et al*. 2012) and *P. aeruginosa* (*wbpJ*) (Penterman *et al*. 2014).

#### Comparison with naturally occurring biofilms

For some of the *in vitro* studies, similar variants could be found in isolates from real-life biofilms. For example, the laboratory-derived SCV (sticky variants) of *P. aeruginosa* described by Kirisits *et al*. (2005) are similar in colony morphology to clinical isolates from a CF lung and also other phenotypes are consistent, including aggregation in liquid culture, hyperadherence and reduced motility. Also for the experimentally evolved *agr* and *sigB* variants in *S. aureus*, clinical isolates containing mutations in the same genes have been isolated (Karlsson-Kanth *et al*. 2006; Traber *et al*. 2008; Savage, Chopra and O'Neill 2013). Furthermore, variants with an altered outer-membrane lipopolysaccharide structure that emerged in an evolution experiment with *P. aeruginosa* have been proposed to also occur in isolates from CF patients (Penterman *et al*. 2014). Indeed, mutations in the same genes were detected both in the variants from the *in vitro* experiment and isolates from CF patients (Warren *et al*. 2011; Davis *et al*. 2013). A final example are the morphological variants that occurred in the *B. cenocepacia* bead transfer model by Poltak and Cooper (2011), which were also found in CF patients isolates (Haussler *et al*. 2003). The same four classes of mutations found in the replicate i*n vitro* populations (c-di-GMP metabolism, LPS gene cluster, Transcription and TCA cycle) were also identified in studies of isolates of *B. dolosa* and *P. aeruginosa* that evolved during CF infections (Smith *et al*. 2006; Cramer *et al*. 2011; Lieberman *et al*. 2011; Traverse *et al*. 2012). In the isolates, many more mutations were observed; however, the amount of mutation overlap was more than expected by chance, which suggests convergent evolution. This parallelism between variation in *in vitro* biofilm evolution experiments and isolates from chronic infections, suggests that adaptation during chronic infections may be at least partly driven by selection in biofilms.

Parallelism has also been observed between mutations in different *in vivo P. aeruginosa* (Smith *et al*. 2006; Cramer *et al*. 2011; Warren *et al*. 2011; Yang *et al*. 2011) and *B. dolosa* (Lieberman *et al*. 2011) isolates from different CF patients. Warren *et al*. (2011) found mutations in different individual isolates that decrease the invasion ability of *P. aeruginosa* in the host and enhance biofilm formation and the occurrence of multidrug efflux pumps. Additionally, both the loss of ability to catabolize 4-hydroxyphenylacetic acid and the increased resistance to ciprofloxacin evolved in individual isolates (Yang *et al*. 2011). Furthermore, Smith *et al*. (2006) and Cramer *et al*. (2011) both found mutations in genes encoding for multidrug efflux pumps, chemotaxis, motility, virulence or QS in different isolates. Finally, transcriptome analysis indicated that change in gene expression between *in vivo P. aeruginosa* isolates also displayed parallelism. Examples include downregulation of pili synthesis and upregulation of biofilm formation (Huse *et al*. 2010) and upregulation of proteins involved in microaerobic growth (Hoboth *et al*. 2009).

### Consequences of diversification

#### The insurance hypothesis

The self-generated diversity during biofilm growth might offer protection against changing and adverse environmental conditions. Populations composed of diverse subpopulations are in general expected to perform better because of the likelihood that some subpopulations will thrive as prevailing conditions change. Monospecies forests are for example more susceptible to environmental perturbations than mixed woodlands (McCann 2000). This principle is known as the ‘insurance hypothesis’ (Yachi and Loreau 1999; McCann 2000; Boles, Thoendel and Singh 2004). In contrast to what the term suggests, the insurance hypothesis does not imply that diversity evolves ‘because’ it helps the population to survive; increased survival is rather a lucky consequence of diversity. Boles *et al*. (2004) found evidence for this process in *P. aeruginosa* biofilms that underwent diversification during growth in different types of biofilm setups. Two main morphotypes (mini and wrinkly) with specialized biofilm phenotypes i.e. accelerated detachment (mini) and hyperbiofilm formation (wrinkly) evolved in these setups through a RecA-dependent mechanism (see above). Although not evolved in the presence of antimicrobials, biofilms inoculated with the wrinkly morphotype alone showed an increased resistance to hydrogen peroxide, hypochlorite and tobramycin. Wild-type biofilms that produced variant subpopulations showed increased resistance to hydrogen peroxide as compared to biofilms formed by the *recA* mutant that did not generate variants. Similarly, Tyerman *et al*. (2013) observed a fast emergence of heritable variation for broad-spectrum antibiotic resistance in *E. coli* biofilms, which were evolved in the absence of antibiotics. Other examples consistent with an insurance effect are Allegrucci and Sauer (2007) and Ryder, Chopra and O'Neill (2012) who found much higher survival of cells isolated from biofilms than planktonic cultures of *S. pneumoniae* and *Staphylococcus* respectively after plating them on antibiotic agar. The occurrence of trade-offs was not directly tested in these study and therefore the term ‘insurance effect’ should be used with caution, as argued by Cooper, Beaumont and Rainey (2005).

Insight into a mechanism by which biofilm diversification might provide an insurance effect was obtained by Koch *et al*. (2014). When grown in colony biofilms, methicillin-resistant *S. aureus* isolates were found to diversify into two new strains. The first strain competed with the parent strain via secretion of a toxic bacteriocin, but the parent strain counter-adapted to this challenge by generating a second new strain which is resistant to the bacteriocin. This same resistance mechanism also provides cross-protection against vancomycin, the prefered antibiotic to treat MRSA. Although these biofilms have not been evolved in the presence of vancomycin, this coevolutionary arms race results in the emergence of a vancomycin resistant variant, which provides an insurance for survival when treatment starts. Importantly, this strain diversification was also shown to occur *in vivo* and both coevolved phenotypes resemble strains commonly found in clinic, emphasizing the relevance of this mechanism (Koch *et al*. 2014).

In sum, evidence suggests that biofilms can serve as hotbeds of diversity that promote adaptation to harsh conditions. Such adaptation could be particularly important in chronic infections inside the host during which bacteria need to withstand severe and fluctuating conditions to persist.

#### Altered biofilm productivity

Another important consequence of diversification is that it can affect total biofilm productivity. Character displacement between coevolving genotypes can optimize the use of available resources and as such increase total productivity (Hector *et al*. 1999; Tilman *et al*. 2001; Bell *et al*. 2005). For example, in the *B. cenocepacia* bead transfer model, increased biofilm productivity was observed over time. As described above, the observed process of ecological succession through niche construction and character displacement coincided with a reduced competition between the evolving variants, the emergence of positive interactions and enhanced community productivity (Poltak and Cooper 2011; Ellis *et al*. 2015). Another example was provided by Brockhurst *et al*. (2006). As described above, in the *P. fluorescens* radiation in static microcosms a WS WS morphotype evolved, which forms a biofilm mat at the air–liquid interface. Brockhurst *et al*. (2006) studied further diversification within this biofilm mat and showed that character displacement between coevolving WS variants takes place within the mat in order to reduce resource competition. As expected, the extent of character displacement between pairs of coevolved WS (measured by the strength of negative frequency-dependent selection) was then found to be positively correlated with the biofilm productivity.

Ecological competition between strains then can cause character displacement, which as a by-product can promote total community productivity. However, this is not guaranteed as character displacement may also reduce productivity of a strain. Moreover, the evolution of interference competition and antibiotic warfare (as exemplified in the previous paragraph (Koch *et al*. 2014)) is likely common and will tend to decrease productivity.

#### Trade-offs between biofilm and free-living state

Another potential consequence of adaptive diversification in biofilms is that evolved cells are no longer adapted to non-biofilm conditions. In a *P. aeruginosa* biofilm evolution experiment, for example, culture-impaired variants arose (Penterman *et al*. 2014). These variants were found to have an altered outer-membrane lipopolysaccharide structure (lack of B-band LPS) compared to the wild type, due to nonsynonymous mutations in *wbpJ*, which increased their fitness within the biofilm but sensitized them to killing by a self-produced antimicrobial outside the biofilm. Proposed explanations of why the mutant has an advantage in biofilm environment were that energy might be saved by not producing B-band EPS or that this leads to an advantage by enhancing the cell's ability to aggregate or adhere. Similar trade-off mechanisms might operate in natural biofilms since it has been shown that in CF infections *P. aeruginosa* evolves mutations inactivating B-band LPS biosynthesis at high frequencies.

These findings suggest that the transition between biofilm growth and free-living state can be costly for bacteria and also raise the possibility that biofilms in natural settings produce large culture-resistant subpopulations that thrive *in situ* but fail to be detected by culture-based sampling (‘viable-but non-culturable’ phenotype). Another example includes the competition-colonization trade-offs between EPS producing and non-producing strains in a *V. cholerae* biofilm. Producers have an advantage in the biofilm because they can move to the top of the biofilm and suffocate their neighboring cells. However, EPS-producers have a decreased dispersion capacity compared to EPS deficient strains (Nadell and Bassler 2011). Local competition is thus dominated by EPS producers, while non-producers have an advantage regarding dispersion capacity. This enhanced ability of non-producers to disperse might explain why certain bacterial species, among which *V. cholerae*, use QS to terminate polymer secretion at high cell density (Nadell *et al*. 2008).

## EVOLUTION OF COOPERATIVE TRAITS IN BIOFILMS

A key outcome of evolution in biofilms that we have not yet discussed is the emergence of cooperative traits. Cooperative traits are phenotypes that increase the fitness of another cell and have at least in part evolved because of this effect (West *et al*. 2006; Mitri and Foster 2013). Simple cooperative traits, such as extracellular enzymes, iron-scavenging siderophores and extracellular polymers, are widespread in the microbial world and in biofilms. Niche specialization is often mediated by the development of traits that are cooperative in nature and as such cooperation plays an important role in adaptive diversification. For example, cellulose overproduction by the WS emerged in the *P. fluorescens* radiation is a cooperative trait, which allows them to form a biofilm mat and obtain—as a group-superior access to oxygen (Rainey and Rainey 2003).

Explaining the evolution of cooperative traits can be challenging because they are prone to exploitation by ‘cheaters’, rapidly growing cell-lines that lack the cooperative trait but benefit from the cooperation of others. There is evidence that cheating occurs within biofilms (Boyle *et al*. 2013). Indeed, as already touched upon above, cellulose production by the WS in the *P. fluorescens* biofilm mat is vulnerable to SM cheaters, which do not produce cellulose but still reap the advantage of residing in the mat. The SM cheaters however disrupt the structural integrity of the mat, which causes it to sink earlier (Rainey and Rainey 2003). Moreover, Popat *et al*. (2012) reported that QS) cheating can occur in *P. aeruginosa* biofilm populations owing to exploitation of QS-regulated public goods. Similarly, Savage, Chopra and O'Neill (2013) reported the emergence of a white variant during *S. aureus* biofilm growth, which due to activation of the *agr* QS system shows an overproduction of a-haemolysin and other extracellular compounds and has an advantage in the early stages of biofilm formation. The presence of a large subpopulation of cells in the biofilm exhibiting enhanced activity of the *agr* system again allowed the emergence of a cheating large pale variant, which is deficient in the QS system and exploits the exoproducts of QS proficient cells.

Social evolution theory, which deals with understanding the evolutionary trajectories of cooperative traits, predicts that, for a given cost-to-benefit ratio of cooperation, the benefits of a cooperative behavior must be sufficiently directed to other cooperating individuals for cooperation to remain evolutionarily stable against exploitation (Hamilton 1964; West *et al*. 2006; Mitri and Foster 2013). That is, cooperation is favored when interaction preferentially occurs between cells that carry the same genotype at the locus driving a social trait, such as clonemates (the genotypic view of microbial interactions; Mitri and Foster 2013).

### Mechanisms that stabilize within genotype cooperation in biofilms

**Figure 3.**
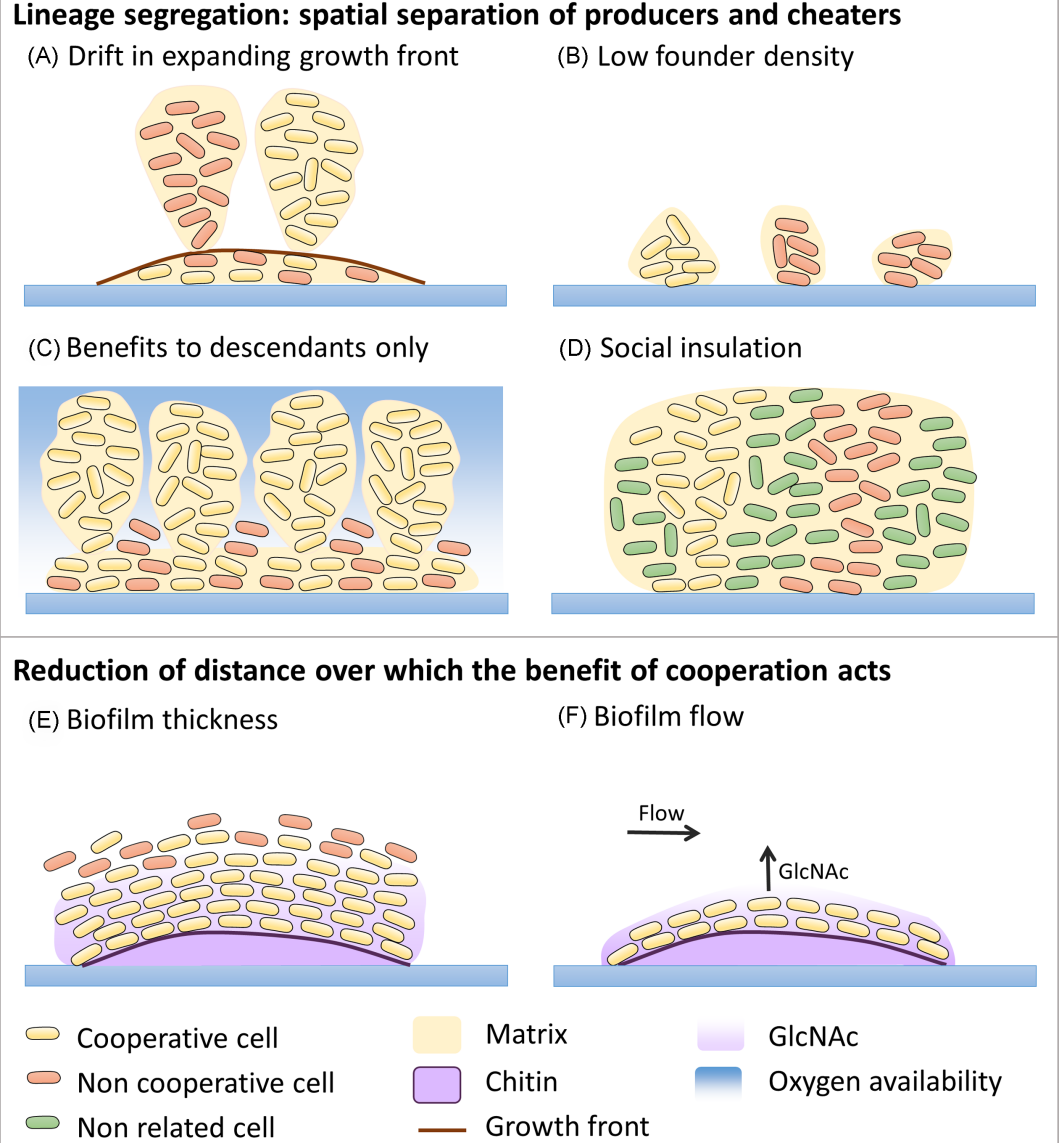
Overview of mechanisms that stabilize cooperation in biofilms. There are two general types of mechanisms: the spatial separation of producers and cheaters (**A**–**D**) and reduction of distance over which the benefit of cooperation acts (**E** and **F**). (A) ‘Drift in expanding growth front’: random drift in the thin active growth layers causes lineage segregation (Nadell, Foster and Xavier 2010). (B) ‘Low founder density’: at low founder cell density, the cells are initially more separated from each other at the surface, allowing more cell divisions before the growing cell clusters get into contact and as such increasing lineage segregation (van Gestel *et al*. 2014). (C) ‘Benefits to descendants only’: polymer production causes the descendants of the producers to be pushed up to areas with an increased oxygen availability, while non-producers are being suffocated (Nadell and Bassler 2011). (D) ‘Social insulation’: at high-nutrient conditions, the addition of another species causes the producers and non-producers to be separated from each other (Mitri, Xavier and Foster 2011). (E) ‘Biofilm thickness’: producers of chitinase (yellow), an enzyme to degrade chitin (blue) to usable GlcNAc, are located at places with a high biofilm density. Due to the thick biofilm, diffusion is low and non-producers (red) are not able take advantage of the GlcNAc as it will be depleted by neighboring producers cells before it can reach the non-producers (Drescher *et al*. 2014). (F**)** ‘Biofilm flow’: under static conditions, the GlcNAc produced from chitin (blue) by chitinase producers (yellow) can also be used by non-producers (red). However, under flow conditions, the GlcNAc will be transported away from the biofilm surface, causing only neighboring producers cells to benefit from the GlcNAc (Drescher *et al*. 2014).

Evolution experiments and competition experiments in biofilms have provided insights in how biofilm growth and structure can reduce the extent of interaction between cooperative and non-cooperative cells and as such promote cooperation (Nadell, Xavier and Foster 2009). Indeed, the ecological diversification we focused on in the last section can help to stabilize cooperation. Diverse groups may be less susceptible to invasion by cheaters, both because it can allow a cooperator to escape the niche of cheat and because a diverse population leaves fewer resources unexploited (Brockhurst *et al*. 2006). Consistent with this Brockhurst *et al*. (2006) found that diverse populations of WS were more resistant to cheating strains than monocultures of WS strains. However, character displacement is just one of several processes in biofilms that can promote cooperation within a genotype. Here, we give an overview of additional processes (Fig. 3). The first four processes act by spatially segregating cooperators and non-cooperator, while the latter two reduce the distance over which the benefit of cooperation acts.

#### Drift in expanding growth fronts favors lineage segregation and cooperation

Biofilm cells often proliferate into larger cell groups. As explained above, experiments with bacterial colonies have indicated that expanding cell groups can segregate into sectors, each dominated by a single genetic lineage (Golding, Cohen and Ben-Jacob 1999; Hallatschek *et al*. 2007). Random genetic drift at the expanding frontiers (i.e. the thin active layer of growing cells at the outside of the colony) is thought to be at the basis of this sectoring (Klopfstein, Currat and Excoffier 2006; Hallatschek *et al*. 2007; Hallatschek and Nelson 2008). By using an agent-based biofilm model that employs mechanistic descriptions of solute diffusion and cell growth, Nadell, Foster and Xavier (2010) showed that the extent of lineage segregation within biofilms is inversely related to the depth of the active layer of growing cells. The active layer depth itself increases with nutrient levels, substrate diffusivity and slower cell growth rate (Mitri, Clarke and Foster 2015). Thick active layers promote lineage mixing, while decreasing active layer depth generates increasingly strong lineage segregation, ultimately leading to the formation of towers consisting of single genotypes.

In order to explore a potential connection between lineage segregation and the evolution of social phenotypes, a cooperative phenotype was incorporated in the model in the form of a diffusible extracellular enzyme that is costly for the cooperative individuals to produce, but benefits all cells in the local area. Competition between a cooperating cell line and an exploitative line was then studied for different active layer depths and thus different levels of segregation. When active layers are thick, leading to well-mixed cell lineages, the enzyme is homogenously distributed through cell groups and the non-cooperative cell line is therefore able to exploit the cooperative line (Nadell, Foster and Xavier 2010). This result is consistent with observations of exploitation in liquid cultures (West *et al*. 2006). With decreasing active layer depth, the cooperative cells and exploitative cells no longer remain well mixed, which results in an increasingly asymmetric distribution of the benefits of the cooperative secreted enzyme to cooperative cells. This allows cooperative cells to outcompete exploitative cells. In summary, this model thus suggests that clusters of genetically related cells can emerge quite easily in spatially constrained cell groups allowing stable persistence of cooperative phenotypes. *In vitro* evidence was provided by Van Dyken *et al*. (2013), who found that expanding colonies of fluorescently labeled *Saccharomyces cerevisiae* cells on agar show genetic demixing of cooperators and cheaters, followed by increase in cooperator frequency as cooperator sectors overtake neighboring defector sectors.

#### Low founder cell density promotes lineage segregation and cooperation

Simulations with an agent-based model revealed that founder cell density also clearly affects spatial segregation within biofilms (van Gestel *et al*. 2014). The degree of spatial segregation is inversely related to the density of cells at the onset of biofilm formation. At low founder cell density, the cells are initially more separated from each other at the surface, allowing more cell divisions before the growing cell clusters get into contact and as such increasing spatial structure. EPS can be considered as a public good as they are shared, provide an advantage to the surrounding cells and are costly to produce. Consistent with the effect of spatial segregation described above, the model predicts that at high founder cell density cooperation by sharing the public good EPS is unstable, while at lower founder cell density EPS producers outcompete non-producers. Similar results were obtained *in vitro* when *B. subtilis* biofilms were inoculated with different inoculum sizes: the lower the inoculum the more spatial segregation and the less exploitation of EPS production.

#### EPS secretion pushes cells up and directs benefit to descendants

An additional mechanism resulting in spatial segregation of cooperators and non-cooperators was described by Xavier and Foster (2007), who applied an agent-based model to study the outcome of evolutionary competitions between strains that differ in their level of EPS production. This study indicated that polymer production is altruistic for cells lying above a focal cell, since it pushes descendants up and out into better oxygen conditions. At the same time, it provides a strong advantage at the scale of the cell lineages by suffocating neighboring non-producers. The upward expansion of the biofilm provides thus a mechanism by which the advantages of polymer production are specifically directed toward the descendants, as such stabilizing cooperation through EPS. In addition, EPS-producing cells better adhere to each other, resulting in strong mother–daughter interactions that may also contribute to stabilization of cooperation (Schluter *et al*. 2015). An interesting side note is that the adhesion properties of EPS can provide additional evolutionary advantages such as resistance to shear stress and an active displacement of non-producing cells near the growth surface. This has been predicted by agent-based models and validated in *in vitro V. cholera* biofilms (Nadell and Bassler 2011; Schluter *et al*. 2015).

#### Social insulation in multispecies biofilms promotes within species cooperation

Metagenomic sequencing has made us aware of the vast level of species diversity associated with biofilms. Mitri, Xavier and Foster (2011) used an agent-based model to study the role of species diversity within biofilm communities on the evolution of social phenotypes, more particularly growth-promoting secretions. At high nutrient concentrations, the additional species were found to act as social insulators that keep non-secretor genotypes away from secretor genotypes and as such promote cooperation within the focal species. Social insulation thus provides a final mechanism causing spatial segregation. Under conditions of strong nutrient competition, however, the addition of new species to a focal species was found to inhibit cooperation within the focal species by eradicating secreting genotypes before they could become established. The potential for ecological competition to preferentially harm cooperators was also shown in an *in vitro* study in which adding *S. aureus* promoted *P. aeruginosa* siderophore non-producers over producers (Harrison *et al*. 2008).

#### Thick biofilms limit diffusion and confine public goods to cooperators

Spatial segregation is not sufficient to stabilize cooperation in all cases. Indeed, Drescher *et al*. (2014) studied factors that could stabilize the costly production of chitinases by *V. cholera*. These are extracellular enzymes that degrade the solid polymer chitin into soluble nutrient-rich GlcNAc oligomers. It was found that biofilm growth of *V. cholerae* on chitin flakes in non-mixed liquid could not completely prevent outcompetition of the wild type by a chitinase non-producer, even not when extending the distance between producers and non-producers by lowering the inoculation size. Strong diffusion effects that homogenize the GlcNAc concentrations and make GlcNAc equally available to non-producers were shown to be at the basis. However, attention was drawn to the spontaneous emergence of matrix hyperproducers that occasionally occurred during these experiments. Matrix hyperproducers were then shown to produce very thick biofilms, in which the public good dilemma was completely solved. In these thick biofilms, cells are multilayered and densely packed, which strongly reduces the diffusion of GLcNAc oligomers because of fast uptake by neighboring cells. As such, by limiting diffusion, production of thick biofilms provides a means to reduce the distance over which the benefit of cooperation acts and to preferentially direct it to cooperators.

#### Fluid flow removes public goods, as such denying access to cheaters

Drescher *et al*. (2014) described an additional mechanism that reduces the distance over which the benefit of cooperation acts. Natural biofilms are often subjected to liquid flow above their surface. It was found that even low flow rates could stabilize chitinase production in *V. cholera* biofilms. Flow facilitates transport of GlcNAc away from the surface of the biofilm, which reduces the concentration available to all cells. This is selectively disadvantageous to chitinase non-producers, because these cells do not benefit from chitinases digesting chitin in their immediate neighborhood. As such, similar to thick biofilms, flow reduces the distance over which the benefit provided by cooperators acts.

### Cooperation among species and strains

Whereas the stabilizing mechanisms explained above focus on cooperation between cells of the same genotype, far less effort has been invested in the study of cooperation between genotypes. The genotypic view of social interactions states that cooperation within genotypes should be common, whereas the conditions for cooperation between genotypes are much more restricted making competition between genotypes the rule (Mitri and Foster 2013). The reason that cooperation within a single genotype should be common is that genetically identical bacterial cells have the same evolutionary interest (Hamilton 1964; West *et al*. 2006; Mitri and Foster 2013). However, different genotypes have their own evolutionary interests. For example, a focal species is only expected to increase the fitness of another species (i.e. evolve cooperative traits) when the return benefits outweigh any costs of ecological competition between the genotypes. Moreover, relatedness within the focal species needs to be high for these return benefits to fall back only on cooperating cells, and not on cheating strains, which would destabilize cooperation.

As described above the emergence of spatiogenetic structure in biofilms is expected to promote cooperation within genotypes (Nadell, Foster and Xavier 2010; Van Dyken *et al*. 2013; van Gestel *et al*. 2014). However, evolutionary models suggest that the effect of spatiogenetic structure on cooperation between genotypes is more complex (Mitri, Xavier and Foster 2011; Oliveira, Niehus and Foster 2014). Indeed, it proves difficult to find conditions that simultaneously favor both within- and among-genotype cooperation. At low nutrient concentrations, spatial segregation prevents the genotypes from interacting with each other. At high nutrient concentrations, genotypic mixing allows the genotypes to interact with each other. However, under the same conditions cheaters also outcompete cooperators. Consistently with the genotypic view, relatively few examples of cooperation between genotypes have been described so far literature. Hansen *et al*. (2007), for example, described the evolution of an exploitative—not cooperative—interaction during biofilm formation, between an *Acinetobacter* strain and a *P. putida* strain that feeds on the benzoate produced by the former. For a more detailed discussion, we refer to Mitri and Foster (2013).

## HOW RELEVANT IS LABORATORY BIOFILM EVOLUTION TO NATURAL CONDITIONS?

An important question is whether observations made for evolution in biofilms in laboratory settings are relevant for evolution in natural biofilms. It can be expected that part of the fast adaptation observed in laboratory evolution experiments is a consequence of growing the bacteria in a novel environment that they have not encountered before. This was illustrated by McElroy *et al*. (2014) who sequenced the metagenome of populations of two *P. aeruginosa* strains (the lab strain PAO1 and the clinical isolate 18A) at two time points during short-term biofilm evolution. Here, it appeared that the better preadaptation of PAO1 to the laboratory environment resulted in a lower number of variants observed when PAO1 was evolved in a biofilm as compared to 18A. It should be noted however that PAO1 only went through half of the number of generations (∼5.3 generations in total) compared to 18A (∼10.3 generations), which could also be a reason for its lower number of variants. Despite these observations, at least two lines of evidence indicate that evolution in laboratory biofilms is not solely adaptation to the laboratory environment and is relevant for natural biofilms. First, many evolution experiments show a much higher level of diversification when the bacteria are grown in biofilms as compared to free-living state, under very similar laboratory conditions. Diversity is quickly lost when the biofilm communities are subjected to planktonic growth (Rainey and Travisano 1998; Boles, Thoendel and Singh 2004; Koh *et al*. 2007; Allegrucci and Sauer 2008; Poltak and Cooper 2011; Savage, Chopra and O'Neill 2013). This indicates that large part of the diversification is a specific consequence of growth under biofilm conditions rather than adaptation to the laboratory environment. Second, the exceptional parallelism found between laboratory-derived variants and *in vivo* isolates indicates that some of the same forces that drive biofilm adaptation *in vitro* also contribute to adaptation during chronic infections (Kirisits *et al*. 2005; Smith *et al*. 2006; Cramer *et al*. 2011; Lieberman *et al*. 2011; Traverse *et al*. 2012; Savage, Chopra and O'Neill 2013; Penterman *et al*. 2014).

## CONCLUSION

Despite the predominance of biofilm growth in nature, only a relatively limited number of evolution experiments have been performed with biofilm populations. Some of these studies employed simple models to identify general principles. For example, a static test tube, called ‘microcosm’, in which *P. fluorescens* is able to diversify and form a biofilm-like mat, has been used in a large number of studies to obtain broad insight into many aspects of the evolutionary process of adaptive radiation (Rainey and Travisano 1998). On the other hand, more complex *in vitro* experiments and even *in vivo* experiments, in which isolates from the same patients were analyzed over time, were performed to gain better understanding of the course of chronic infections and persistent contaminations per se, and to aid the design of improved therapeutics and disinfectants.

The majority of biofilm evolution studies have focused on the fast emergence of morphotypic, phenotypic and genotypic variation within biofilms. Several evolutionary and ecological processes have been proposed to underlie this diversification, although the relative role of each of them remains to be determined and is likely strongly dependent on the nature of the specific biofilm under study. First, the environmental heterogeneity within biofilms provides ecological opportunity, which can result from one strain modifying the environment in a manner that allows another to thrive (Poltak and Cooper 2011). Diversifying selection, generated by resource competition, favors the emergence of diverse ecological niche specialists. Additionally, biofilm structure can subdivide the population into a number of more or less independently evolving subpopulations, a phenomenon called population fragmentation, which may promote the fixation of diverse mutations of smaller effect through an increased influence of drift and a lower access to rare beneficial mutations of large effect (Habets *et al*. 2006; Hallatschek *et al*. 2007). Similarly, the presence of gradients may provide stepping stones for diverse mutations of smaller effect. Another source of diversification in biofilms comes from the increased fixation time associated with clonal interference, which itself is a consequence of the slow wave-like spread of beneficial mutations through space in structured populations (Martens and Hallatschek 2011). Finally, biofilms experience an increased level of HGT and an increased mutation rate through (oxygen) stress induced mutagenesis and the occurrence of hypermutator phenotypes. These provide the genetic variation for drift and diversifying selection to act upon.

A growing body of knowledge is being generated on the genetic mechanisms underlying diversification within biofilms. The evolution of a stable, diverse community requires the occurrence of fitness trade-offs between niches, so that no single genotype is able to outcompete the others in all niches. Analysis of variants of the *P. fluorescens* radiation in static microcosms indicated antagonistic pleiotropy, i.e. alleles beneficial in one environment being deleterious in others, as an important molecular mechanism behind this fitness trade-offs across niches (MacLean, Bell and Rainey 2004). Intriguingly, Traverse *et al*. (2012) found, through metagenome sequencing of their diversifying *B. cenocepacia* biofilm population, that competition between genotypes cannot only act within niches, but that recurrent invasion of niches by novel genotypes originated in other niches is possible as well.

A striking amount of parallelism has been observed in biofilms at the morphotypic, phenotypic and genotypic level, both between replicate lineages within the same evolution experiment and across different evolution experiments. Moreover, the exceptional parallelism found between laboratory-derived variants and *in vivo* isolates indicates that some of the same forces that drive biofilm adaptation also contribute to adaptation during chronic infections (Traverse *et al*. 2012; Lind, Farr and Rainey 2015).

Biofilm diversification has important consequences for bacterial survival and productivity and its study is therefore of key importance to design improved antimicrobial strategies and diagnostic techniques. First, the self-generated diversity during biofilm growth might offer protection against changing and adverse environmental conditions, a principle known as the ‘insurance hypothesis’ (Boles, Thoendel and Singh 2004). Most worrisome, it has been shown that biofilm diversification can lead to increased antibiotic resistance, even when strains evolve in the absence of antibiotics (Koch *et al*. 2014). Additionally, diversification can increase biofilm productivity because of a more efficient use of all available resources (Ellis *et al*. 2015). Finally, some of the evolved variants can experience stark trade-offs with opposing effects on fitness inside and outside biofilms (Penterman *et al*. 2014). This might result in biofilm subpopulations which are non-culturable under standard laboratory conditions, and as such impede diagnostics.

Diversification in biofilms can also stabilize cooperative traits, not in the least because diverse groups are likely less susceptible to invasion by non-cooperative cheaters (Brockhurst *et al*. 2006). More generally, there appear to be many routes to the evolution of cooperation within biofilms, particularly between cells of the same genotype that have the same evolutionary interests (Hamilton 1964; West *et al*. 2006; Mitri and Foster 2013). Evolution experiments have provided insights in how biofilm growth and structure can reduce the extent of interaction between cooperative and non-cooperative cells and as such promote cooperation (Nadell, Xavier and Foster 2009). These strategies either act by spatially segregating cooperators and non-cooperator, or by reducing the distance over which the benefit of cooperation acts. First, random genetic drift at the active layer of growing cells can favor segregation between cooperating and non-cooperating lineages, as such reducing the interaction and stabilizing the cooperation between both (Nadell, Foster and Xavier 2010; Van Dyken *et al*. 2013). Similar lineage segregation can be caused by inoculating strains at low density (van Gestel *et al*. 2014). Indeed, at low founder cell density, the cells are initially more separated from each other at the surface, allowing more cell divisions before the growing cell clusters get into contact.

The evolution of EPS production can push each other up into better oxygen conditions. The upward expansion of the biofilm provides a mechanism by which EPS producers are segregated from non-producers and the advantages of EPS production are specifically directed toward the descendants (Xavier and Foster 2007). Moreover, EPS producing cells adhere better to each other, resulting in strong mother–daughter interactions that may also contribute to stabilization of cooperation (Nadell and Bassler 2011; Schluter *et al*. 2015). Social insulation by additional genotypes in multispecies biofilms provides a final mechanism causing spatial segregation to keep non-secretor genotypes away from secretor genotypes and as such promote cooperation within the focal species (Mitri, Xavier and Foster 2011). Another strategy to stabilize cooperation is the production of thick biofilms, since this can limit the diffusion of shared extracellular products and as such confine these public goods to the cooperators (Drescher *et al*. 2014). Similarly, fluid flow above the biofilm can direct public goods to cooperators by rapidly removing the public goods, as such denying access to cheaters (Drescher *et al*. 2014). The genotypic view of social interactions predicts that cooperation between genotypes is much less common because the focal genotype is expected to increase the fitness of another genotype (i.e. evolve cooperative traits) only when the return benefits outweigh any costs of ecological competition between the genotypes (Mitri and Foster 2013).

Whereas most of the biofilm evolution studies up till now focused on the analysis of characteristics and interactions of a few isolated variants at the endpoint of the experiment, the advent of whole-genome and whole-population sequencing techniques (Barrick and Lenski 2013), facilitated by improving haplotype reconstruction protocols (Pulido-Tamayo *et al*. 2015), will undoubtedly lead to an explosion of information on many aspects of evolutionary dynamics in biofilms in the coming years. Moreover, the continually improving techniques to precisely isolate and sequence single cells and to study gene expression at the single cell level *in situ* will make it possible to accurately correlate specific genotypes with specific biofilm niches. For a detailed overview of upcoming techniques to study genomes and gene expression at the single cell level we refer to our recent review on this topic (Roberfroid, Vanderleyden and Steenackers 2016).

Whereas most research on biofilm evolution until now has been centered around mechanisms of diversification and cooperation, our analysis of the literature reveals that at least two research topics deserve increasing attention in the future. A first topic is development of resistance against antimicrobials in biofilms. Indeed, it can be envisioned that several of the ecological and evolutionary processes behind diversification can also promote a faster resistance development against antimicrobials, especially the higher mutation rate and HGT within biofilms and the stepping stones provided by gradients of antimicrobials. Moreover, biofilm growth might promote the evolution of cooperative resistance mechanisms, such as extracellular enzymes that degrade antibiotics, which are not stable in planktonic cultures. Second, the vast majority of studies focused on diversification or cooperation within monospecies biofilms, whereas evolution within more realistic multispecies biofilms is a major area that remains to be explored.

## Supplementary Material

Supplementary Data
