# Supplementary material for: Experimental evolution in biofilm populations
Source: FEMS Microbiol Rev. 2016 Feb 18;40(3):373–97. doi: 10.1093/femsre/fuw002 (PMC4852284; doi:10.1093/femsre/fuw002)
Supplement: Supplementary Data [file fuw002_supplementary_data.zip › Supplementary_table_1.docx]

**Supplementary table 1. Overview of experimental models used to study evolution in biofilms**

| **Model** | **Description of model** | **Focus of study** | **Reference** |
| --- | --- | --- | --- |
| Static microcosm of *P. fluorescens* | Non shaking test tube, in which a biofilm mat forms at the broth-air interface | Effect of ecological opportunity on diversification | Rainey & Travisano (1998) |
|  |  | Relationship between productivity and diversity through its effect on the relative production of niches | Kassen *et al.* (2000) |
|  |  | Relation between disturbance and diversity | Buckling *et al.* (2000) |
|  |  | Genetic and phenotypic causes of the evolution of the Wrinkly Spreader (WS) phenotype | Spiers *et al.* (2002) |
|  |  | Role of the *wss* operon and the acetylation of cellulose in the formation of the WS phenotype | Spiers *et al.* (2003) |
|  |  | Evolution of cooperation in a spatially heterogeneous environment | Rainey & Rainey (2003) |
|  |  | Effect of level of resource input (productivity) and environmental disturbance on diversification | Kassen & Rainey (2004) |
|  |  | Ecological constraints on diversification | Kassen *et al.* (2004) |
|  |  | Metabolic cost associated with the evolution of the WS phenotype | MacLean *et al.* (2004) |
|  |  | Role of LPS in providing strength to the WS biofilm mat | Spiers & Rainey (2005) |
|  |  | Role of WspR in the evolution of the Large Spreading Wrinkly Spreader (LSWS) morphotype | Goymer *et al.* (2006) |
|  |  | Stabilization of cooperation due to adaptive diversification | Brockhurst *et al.* (2006) |
|  |  | Effects of a single point mutation on proteome differences between Smooth Morphology (SM) and LSWS phenotype | Knight *et al.* (2006) |
|  |  | Characterization of the Wsp pathway to understand diversity within the LSWS morphotype | Bantinaki *et al.* (2007) |
|  |  | Influence of bottleneck size on the evolution of cooperation | Brockhurst (2007) |
|  |  | Effect of disturbances on cooperation | Brockhurst *et al.* (2007) |
|  |  | Effects of residence population on the diversification of an invading population | Brockhurst *et al.* (2007) |
|  |  | Identification of Aws and Mws as important genes in the evolution of the WS phenotype and explanation why parallel evolution follows a subset of all possible pathways | McDonald *et al.* (2009) |
|  |  | Mechanism of loss of diversity during later stages of adaptive radiation | Meyer *et al.* (2011) |
|  |  | Genetic and phenotypic causes of the evolution of the Fuzzy Spreader (FS) phenotype | Ferguson *et al.* (2013) |
|  |  | Review of the mechanistic causes of the evolution of the WS phenotype | Spiers (2014) |
|  |  | Genetic basis of how parallel evolution is biased towards a subset of pathways and identification of new pathways leading to the WS phenotype | Lind *et al.* (2015) |
| Bead transfer model of *B. cenocepacia* | In slowly rotating test tubes, biofilms are formed on plastic beads, which are regularly transferred to new test tubes. Cells must disperse and colonize a new bead in order to be transferred. | Long-term evolution and diversification of *B. cenocepacia* and quantification of fitness of evolved variants | Poltak & Cooper (2011) |
|  |  | Reconstruction of evolutionary dynamics of long-term adaptation of one *B. cenocepacia* lineage | Traverse *et al.* (2012) |
|  |  | Mutational and ecological causes of the evolution of the Wrinkly (W) phenotype | Cooper *et al.* (2014) |
|  |  | Link between the succession of genotypes and changing interactions | Ellis *et al.* (2015) |
| Spotting on solid agar plates | minimal salt agar plates, with 2,4 D | The effect of a structured environment on the phenotypic diversification of *Comamonas* sp. | Korona *et al.* (1994) |
|  | minimal medium, with glucose | The effect of spatial structure on the rate of adaptation during evolution in *E. coli* | Perfeito *et al.* (2008) |
|  | 2X SG plates | Effect of density of founder cells populations of *B. subtilis* on spatial segregation and evolution of cooperative phenotypes | van Gestel *et al.* (2014) |
|  | LB plates | Phenotypic diversification in *E. coli* single colonies and the underlying causes | Saint-Ruf *et al.* (2014) |
|  |  | Genetic segregation and drift in *E. coli* colonies | Hallatschek *et al.* (2007) |
|  | Pseudomonas agar plate F | Competition between evolved variants under spatial heterogeneous conditions | Kim *et al.* (2014) |
|  | Tryptic Soy Broth agar with magnesium added | Competition between variants and the emergence of antibiotic resistant *S. aureus* strains | Koch *et al.* (2014) |
|  | Minimal and rich medium on structure intact (transferred by a stamp) and mixed plates (mixed before transferring on new plate) | Effect of population structure on the emergence and persistence of diversification of *E. coli* | Habets *et al.* (2006) |
| Non-shaking microtiter plate | Biofilms are grown on the bottom or on discs on the bottom of the wells | Adaptive evolution of *E.coli* biofilms and fitness increase of evolved variants compared to the wild type | Kraigsley & Finkel (2009) |
| Cellulose disk static (CDS) model | Biofilms are grown on cellulose disks, soaked in human plasma | Diversification in *S. aureus* with a static and continuous flow model and genetic analysis of variants | Savage *et al.* (2013) |
| Sorbarod model | Biofilms grow on cylindrical filters composed of compacted cellulose fibres |  |  |
| Tube reactor | Biofilm grows on silicone tubes through which medium flows | Analysis of morphotypic variants that evolved in *P. aeruginosa* biofilms | Kirisits *et al.* (2005) |
| Tube reactor | Biofilm grows on silicone tubes through which medium flows | Diversification in *P. aeruginosa*, using different models and explanation of why this diversity occur | Boles *et al.* (2004) |
| Rotating disk reactor | Biofilms are grown on removable coupons that rotate in the medium in the reactor |  |  |
| Drip flow reactor | Medium is continuously dripped on the bacterial growth surface |  |  |
| Non-shaking microtiter plate | Biofilms are grown on the bottom or on discs on the bottom of the wells |  |  |
| Drip flow reactor | Medium is continuously dripped on the bacterial growth surface | Trade off in fitness in evolved variants in *P. aeruginosa* biofilms. | Penterman *et al.* (2014) |
|  |  | Emergence of variants in *S. aureus* that differ in virulence factors, caused by Agr mutations | Yarwood *et al.* (2007) |
| Flow cell | Biofilms experience flow conditions | Evolution of interactions between *P. putida* and *Acetobacter sp.* | Hansen *et al.* (2007) |
|  |  | Timely evolution of variants in *S. marcescens* | Koh *et al.* (2007) |
|  |  | Effect of a mutator phenotype on diversification in *P. aeruginosa* | Lujan *et al.* (2011) |
|  |  | Genetic analysis of short term diversification in *P. aeruginosa* | McElroy *et al.* (2014) |
|  |  | Evolution and diversification in *E. coli* biofilms | Ponciano *et al.* (2009) |
|  |  | Solution to how public goods and cooperation can remain evolutionary stable in a *V. cholera* biofilm. | Drescher *et al.* (2014) |
|  |  | Evolution of antibiotic resistance in structured environments | Zhang *et al.* (2011) |
| Flow cell | Biofilms experience flow conditions | Morphologic, phenotypic and molecular analysis of variants that evolved in *S. pneumoniae* biofilms | Allegrucci & Sauer (2007) |
| Non-shaking microtiterplate | Biofilms are grown on the bottom or on discs on the bottom of the wells |  |  |
| Spotting on CF sputum model | Use of CF sputum as growth media | Genetics and adaptation of *P. aeruginosa* to CF like environment, in presence or absence of ciprofloxacin | Wong *et al.* (2012) |
| Soil model | Use of soil as growth media | Adaptation of *P. fluorescens* to a soil environment, in presence or absence of a resident community | Gomez & Buckling (2013) |
| *In silico* models | Mathematical modeling of biofilms | Cooperation and competition between strains that differ in their level of polymer production | Xavier & Foster (2007) |
|  |  | Variation in activating or terminating polymer secretion by the QS system when reaching high cell densities in *V. cholerae* | Nadell *et al.* (2008) |
|  |  | Development of a model to give an explanation for empirically observed cooperation under spatially heterogeneous conditions | Xavier *et al.* (2009) |
|  |  | Evolution of cooperation under spatially structured conditions | Nadell *et al.* (2010) |
|  |  | Evolution of cooperative secretion in the presences of two different species and under different nutrient availabilities | Mitri *et al.* (2011) |
|  |  | The role of adhesion in biofilm evolution | Schluter *et al.* (2015) |
| *In vivo* models | Biofilm isolates from CF patients | Analysis of the genetic adaptation during coevolution of *P. aeruginosa* and its human host by studying several patient isolates. | Smith *et al.* (2006) |
|  |  | Transcriptomic profiling of *P. aeruginosa* strains, isolated during 39 000 generations *in vivo* by studying several patient isolates. | Huse *et al.* (2010) |
|  |  | Evolution of QS and virulence in *P. aeruginosa* isolates, collected over 20 days from 31 patients | Kohler *et al.* (2009) |
|  |  | Importance of phenotypic and genotypic variants of *P. aeruginosa* that evolved during in vivo evolution. | Warren *et al.* (2011) |
|  |  | Characterization of evolutionary dynamics of *P. aeruginosa* in its human host over 200 000 generations by studying several patient isolates. | Yang *et al.* (2011) |
|  |  | Microevolution of the two major clonal complexes of *P. aeruginosa* during infections in lungs of CF patients. | Cramer *et al.* (2011) |
|  |  | Identification of recurring patterns in the evolution of a single *B. dolosa* strain in CF patients. | Lieberman *et al.* (2011) |
|  |  | Characterize adaptive changes in the physiology of *P. aeruginosa* during chronic lung infection in CF patients using transcriptome and proteome analysis. | Hoboth *et al.* (2009) |
